# Transcriptome Reprogramming of Tomato Orchestrate the Hormone Signaling Network of Systemic Resistance Induced by *Chaetomium globosum*

**DOI:** 10.3389/fpls.2021.721193

**Published:** 2021-09-23

**Authors:** Jagmohan Singh, Rashmi Aggarwal, Bishnu Maya Bashyal, K. Darshan, Pooja Parmar, M. S. Saharan, Zakir Hussain, Amolkumar U. Solanke

**Affiliations:** ^1^Fungal Molecular Biology Laboratory, Division of Plant Pathology, ICAR—Indian Agricultural Research Institute, New Delhi, India; ^2^Forest Protection Division, IC FRE-Tropical Forest Research Institute, Jabalpur, India; ^3^Division of Vegetable Science, ICAR—Indian Agricultural Research Institute, New Delhi, India; ^4^ICAR-National Institute for Plant Biotechnology, ICAR-IARI, New Delhi, India

**Keywords:** ISR, SAR, *Chaetomium globosum*, RNA seq, tomato

## Abstract

*Chaetomium globosum* is a potential biological control agent effective against various plant pathogens. Several reports are available on the mycoparastism and antibiosis mechanisms of *C. globosum* against plant pathogenic fungi, whereas a few states induced resistance. The potential induced defense component of *C. globosum* (Cg-2) was evaluated against early blight disease of tomato (*Solanum lycopersicum)* and further, global RNA sequencing was performed to gain deep insight into its mechanism. The expression of marker genes of hormone signaling pathways, such as *PR1, PiII, PS, PAL, Le4*, and *GluB* were analyzed using real-time quantitative reverse transcription PCR (qRT-PCR) to determine the best time point for RNA sequencing. The transcriptome data revealed that 22,473 differentially expressed genes (DEGs) were expressed in tomato at 12 h post Cg-2 inoculation as compared with control plants and among these 922 DEGs had a fold change of −2 to +2 with *p* < 0.05. The Kyoto Encyclopedia of Genes and Genomes (KEGG) pathway analysis revealed that most of the DEGs were belonging to metabolic pathways, biosynthesis of secondary metabolites, plant–pathogen interaction, chlorophyll metabolism, and plant hormone signal transduction. Gene Ontology (GO) analysis revealed that DEGs were enriched mainly related to binding activity (GO:0005488), catalytic activity (GO:0003824), metabolic process (GO:0008152), cellular process (GO:0009987), response to stimulus (GO:0050896), biological regulation (GO:0065007), and transcription regulator activity (GO:0140110). The gene modulations in hormone signaling transduction, phenylpropanoid biosynthesis, and mitogen-activated protein kinases (MPK) signaling indicated the upregulation of genes in these pathways. The results revealed active participation of jasmonic acid (JA) and salicylic acid (SA) signaling transduction pathways which further indicated the involvement of induced systemic resistance (ISR) and systemic acquired resistance (SAR) in the systemic resistance induced by Cg-2 in tomato.

## Introduction

*Chaetomium globosum* is an important biocontrol agent reported to be effective against a number of soil, seed, and airborne fungal diseases in plants, such as spot blotch of wheat, rice blast, root rot of citrus, potato dry rot, and late blight of potato (Aggarwal et al., 2004; Park et al., 2005; Shanthiyaa et al., 2013; Quyet et al., 2016; Jiang et al., 2017). The antagonistic mechanisms of *C. globosum* comprise of competition for space and nutrients, mycoparasitism, and metabolite production (antibiosis), such as prenisatin, chaetoglobosin, chaetocin, cochliodinol, and chaetomin (Brewer et al., 1972; Brewer and Taylor, 1978; Vannacci and Harman, 1987; Soytong, 1992). Antibiosis and mycoparasitism have been observed against various fungal pathogens, such as *Bipolaris sorokiniana, Setosphaeria turcica*, and *Phytophthora palmivora* (Aggarwal et al., 2011; Zhang et al., 2013; Hung et al., 2015). Further, under *in vitro* conditions *C. globosum* inhibits the mycelial growth of *Puccinia triticina* and *Bipolaris sorokiniana* in wheat (Aggarwal et al., 2016). Previous studies stated that among six isolates of *C. globosum*, two isolates (Cg-1 and Cg-6) showed mycoparasitism, whereas, other four isolates (Cg-2, Cg-3, Cg-4, and Cg-5) showed antibiosis against *Cochilobolus sativus*. The potential Cg-2 showed high antifungal metabolite production and gave maximum bio-efficacy under laboratory and glasshouse conditions (Aggarwal et al., 2004). A plethora of information is available on mycoparasitism and antibiosis as a biocontrol mechanism of *C. globosum*, whereas the induced resistance component is least explored (Aggarwal, 2015).

Systemic resistance induced in plants against phytopathogens is broadly categorized as systemic acquired resistance (SAR) and induced systemic resistance (ISR). SAR is generally activated in plants during plants and necrosis inducing pathogen interactions and orchestrated through salicylic acid (SA) mediated signaling pathways (Ryals et al., 1996; Durrant and Dong, 2004). Interaction of rhizobacteria and beneficial fungi with plant rhizosphere also induces a similar systemic resistance in above-ground parts known as ISR. Similar to SAR, the ISR defense mechanism is mediated by jasmonic acid (JA) and ethylene (ET) signaling pathways, independent of SA (Pieterse et al., 1998).

A few studies have been reported highlighting the importance of the induced resistance mechanism exhibited by *C. globosum* toward phytopathogens. For instance, the pretreatment of wheat seed with crude extract of *C. globosum* induced resistance in seedlings against spot blotch (*B. sorokiniana*) (Biswas et al., 2012). It is also reported that *C. globosum* and its metabolites have the ability to induce host defense against tan spot of wheat incited by *Pyrenophora tritici-repentis* (Istifadah and McGee, 2006). Substantial research has been performed in predominant biocontrol agent *Trichoderma* spp. inducing resistance in plants against various fungal phytopathogens using transcriptomic and proteomic approaches. Previous studies state that *Trichoderma asperellum* treatment in *Arabidopsis* induces the ISR against *Pseudomonas syringae*, independent of SA, as the SA-impaired mutant *sid2* expressed ISR (Segarra et al., 2009). In contrast to *T. asperellum, T. longibrachiatum* H9 showed induced systemic resistance in cucumber against *B. cinerea* by the activation of JA/ET and SA pathways (Yuan et al., 2019). Hence, to gain insights into the potential induced defense mechanism of *C. globosum* in tomato (*Solanum lycopersicum*) against early blight disease, global RNA sequencing of tomato plants pretreated with potential Cg-2 strain is performed in this study.

Tomato (*S. lycopersicum*) plant was chosen for this study as it is a model plant and an important commercial crop with significant availability of data of complete genome and reference transcriptome in online genome databases, such as Sol Genomics Network (SGN), Tomatomics, Tomato Genomic Resources Database (TGRD), Plant Genome and Systems Biology (PGSB) Tomato Genome Database, Micro-Tomato Database (MiBASE), and Kazusa Full-Length Tomato (KafTom) Database, etc. (Suresh et al., 2014). A necrotrophic foliar disease, early blight of tomato incited by *Alternaria solani* was taken for evaluating the systemic resistance in tomato induced by seed priming and soil drenching with Cg-2. A foliar disease was selected to spatially separate the soil drenched Cg-2 from *A. solani* to rule out the antagonism and mycoparasitism mechanism of the biocontrol agent. After the evaluation of induced systemic resistance, in the next experiment, the molecular mechanism of resistance induced by *C. globosum* in tomato was explored by transcriptome profiling of Cg-2 treated plants vs. untreated plants and validated by using real-time quantitative reverse transcription PCR (qRT-PCR). The transcriptomic approach provides the complete view of differentially expressed genes under various conditions; therefore, it proved useful for getting insight into the molecular mechanism of induced resistance by visualizing the genes differentially expressed in Cg-2 treated plants as compared with the untreated plants.

## Materials and Methods

### Plant Material and Fungal Cultures

Tomato seeds of the variety Pusa Rohini were procured from the Division of Vegetable Science, ICAR-Indian Agricultural Research Institute, New Delhi. Tomato seeds (10 g) were sterilized with 1% (v/v) sodium hypochlorite followed by three times washing with sterilized distilled water. The seeds were dried in shade and sown at 0.5-inch depth in 12-inch plastic pots filled with sterilized sand:soil (3:1). Twenty-one-day-old seedlings were transplanted in the 6-inch plastic pots with 1–2 seedlings per pot in a polyhouse. Fungal culture of *Alternaria solani* was procured from Indian Vegetable Research Institute, Varanasi, India; sub-cultured on PDA media and incubated at 25°C (16 h light and 8 h dark) in a biochemical oxygen demand (BOD) incubator. The potential biocontrol strain Cg-2 of *C. globosum* isolated in New Delhi from wheat leaf surface (ITS accession no. AY429049) (Aggarwal et al., 2004) was used (ITCC accession no. 6210) for the entire study.

### Biocontrol Agent and Pathogen Inoculation

The biocontrol treatment of tomato plants consisted of application of double dose of Cg-2 first as seed treatment and second dose as drenching of soil with Cg-2 spore suspension (1 × 10^6^ spores per ml) @ 100 ml per pot at 3–4 leaf stage of the plant (as per preliminary experiments). The Cg-2 treated, and untreated plants were counter-inoculated with *A. solani* (As) by spraying suspension after 24 h of Cg-2 application of Cg-2 spore suspension. The plants were placed at 28–30°C and >80% relative humidity for 5 days. This experiment setup included two treatments, T1 as untreated plants counter inoculated with *A. solani* and T2 as Cg-2 treated plants counter inoculated with *A. solani*. Fifteen replications were maintained for each treatment considering a single plant as a replicate.

### Evaluation of the Effect of *C. globosum* Induced Systemic Resistance Against Early Blight Disease of Tomato

The inoculated plants were scored for disease severity using the 0–5 scale (Pandey et al., 2003) at three time points: first observation for the disease severity was taken at 7 days after *A. solani* inoculation and subsequently, observations were taken at 14 and 21 days after inoculation. The disease severity was further utilized to calculate the percentage disease index (PDI) and the area under disease progress curve (AUDPC) following the methodology of Campbell and Madden (1990), Johnson and Wilcoxson (1980), and Van der Aplank (1963), respectively.


$$\begin{array}{r} \text{PDI }=\;\frac{\text{sum of all ratings }\times \;100}{\text{total no}.\text{ of observations }\times \text{ maximum rating grade}}\\ \text{AUDPC }=\;\overset{n-1}{\underset{i=1}{\sum }}\left[\left(\frac{X_{i+1}\;+\;X_{i}}{2}\right)\;\times \;\left(t_{i+1}\;-\;t_{i}\right)\right] \end{array}$$

where *X*_*i*_ is PDI at the *i*th observation, *t*_*i*_ is time (in days after As inoculation) at the *i*th observation, and *n* is the total number of observations.

### Effect of *C. globosum* on Tomato Plant Growth Promotion

The biocontrol treated plants were analyzed for shoot and root length to determine the effect of *C. globosum* on different growth parameters of tomato plants. The pot experiment was designed based on the completely randomized design (CRD) setup that consisted of two treatments: T1 (Cg-2 untreated plants) and T2 (Cg-2 treated plants), with 20 plants for each treatment, and each plant as one replicate. The plant height was recorded for 3 months and root length was also measured. Further, observations were taken for the physiological parameters, such as stomatal conductance (*gs*), photosynthesis rate (*P*_*N*_), and transpiration rate (*E*) by using IRGA LI-6400XT portable photosynthesis system (Lincoln, NE, USA) for 4 months old plants. The differences between means of the treatments for the different growth and physiological parameters were tested using one-way ANOVA and using Student's *t*-test. The differences were considered statistically significant at *p* < 0.05. A statistical analysis of physiological data was performed using SPSS software (IBM SPSS Statistics version 27.0, IL, USA).

### Experiment Setup for Collection of Samples for Transcriptomics

After evaluation of systemic induced resistance by Cg-2 in tomato in experiment 1, another experiment was set up for transcriptome sampling. The experiment setup consisted of two treatments: T1 (Cg-2 untreated plants) and T2 (Cg-2 treated plants). The Cg-2 treatment was given in double dose as mentioned earlier. The foliar samples were collected from the untreated plants and biocontrol treated plants at five time points [6, 12, 24, 48, and 96 h post Cg-2 inoculation (hpCi)] after application of Cg-2 spore suspension with two replications for each and stored at −80°C.

### RNA Extraction

The total RNA was isolated from the six plant samples with two replications (control plants mock-inoculated with sterilized water; biocontrol treated plants at five-time intervals) using trizol and following the guidelines of the manufacturer. The leaf samples (100 mg) were ground with pestle-mortar using liquid nitrogen, transferred to a 1.5 ml eppendorf tube, homogenized with 1 ml trizol. The homogenate was kept at 25°C for 5 min and a 200 μl of chloroform was added to each tube followed by incubation at 25°C after vertexing. The samples were phase-separated centrifuge at 12,000 rpm for 15 min (Eppendorf AG, *Heidelberg, Germany*) and the transparent aqueous phase at the top was transferred to fresh tubes. Later, 500 μl of isopropanol was added to each tube and incubated at room temperature (RT) for 5 min. The samples were then centrifuged at 12,000 rpm for 10 min to obtain an RNA pellet. The pellet was washed with 75% ethanol (v/v) three times by intermittent centrifugation at 7,500 rpm for 5 min. The RNA pellet was air dried for 30 min to evaporate residual ethanol. Then, 40 μl of nuclease free water was used to dissolve the pellet in and incubated in a water bath at 55°C. The RNase-free DNase was used in removing the residual DNA for 30 min at 37°C. The RNA samples were quality checked and quantified by using the NanoDrop (Thermo Fisher Scientific, Wilmington, DE, USA).

### Gene Expression Analysis of Marker Genes of Hormone Signaling Pathways by qRT-PCR

The expression patterns of some characterized marker genes of hormone signaling pathways were analyzed to select the most significant time point (post Cg-2 inoculation) for RNA-sequencing. The six marker genes of plant hormone signaling pathway were selected which included *PR1* and *PAL* for SA pathway (Klessig et al., 2018), *MC* and *PiII* for JA pathway (Andreou et al., 2009), *Glu* for ET pathway, and *Le4* for abscisic acid (ABA) pathway. Primers were designed for these marker genes by using gene script qRT-PCR primer designer (https://www.genscript.com/tools/real-time-pcr-taqman-primer-design-tool). All the qRT-PCR reactions for the marker genes were performed at all the time points, i.e., 6, 12, 24, 48, and 96 hpCi with 0 hpCi as control and each sample with six replicates (three biological replicates × two technical replicates). For gene expression analysis, RNA was converted to cDNA by using a cDNA synthesis kit (Thermo Fisher Scientific Verso). A 2 μg of RNA was used for 20 μl reaction of cDNA synthesis and protocol was performed as per the guidelines of the manufacturer. Each reaction of 20 was prepared with: 4 μl of a 5x cDNA synthesis buffer, 2 μl of a 20 mM dNTP mix, 1 μl of an anchored oligo dT (500 ng/μL), 1 μl of an RT enhancer, 1 μl of a Verso enzyme mix, 2 μg of RNA template, and volume made up to 20 μl with nuclease-free water. The reaction mix was spun down and kept in a thermocycler at 42°C for 45 min followed by the inactivation of reverse transcriptase enzyme by incubating at 95°C for 2 min.

The qRT-PCR reaction mix was preformed using the specific primer pairs (Supplementary Table 1), and elongation factor (*SlEF*) was used as an internal control (Rotenberg et al., 2006). The 20 μl reaction was prepared by using 10 μl of SYBR Green PCR master mix (Thermo Fisher Scientific, MA, USA), 0.5 μl of a forward primer (1 pM), and 0.5 μl of a reverse primer (1 pM) with 1 μl of cDNA as template and with nuclease-ree water final volume was made 20 μl. The qRT-PCR machine (CFX96 Touch Real-Time PCR system, Biorad, Hercules, CA, USA) was used to perform PCR reaction. The PCR conditions were set as: an initial step at 94°C for 4 min, the 40 cycles consisted of 94°C for 15 s, 57°C for 30 s, and 70°C for 30 s followed by dissociation at 72°C for 1 min, and from 75 to 90°C with a rise by 1°C every 5 s to obtain melt curves. The relative gene expression was calculated in terms of fold changes using the 2^−ΔΔCt^ method (Kenneth and Thomas, 2001, Singh et al., 2020). The results are expressed as arithmetic means and SDs of six replicates.

### RNA Sequencing (RNA Seq)

Based on the marker gene expression analysis, the specific time point was identified for the RNA Seq for four tomato samples that included Cg-2 inoculated plants at 12 hpCi and control plant (mock-inoculated with water) and two replicates for each sample. The mRNA enrichment was performed by using poly-T attached magnetic beads, followed by enzymatic fragmentation. The paired-end (PE) sequencing libraries were prepared by using the Illumina TrueSeq stranded mRNA Sample Preparation Kit (Illumina Inc., San Diego, CA, USA) (Kaur et al., 2021). The purification of ds cDNA samples was performed by using Ampure XP beads, followed by A-tailing, adapter ligation, and further enrichment by PCR. The effective concentration of the library was then precisely quantified using a qRT-PCR to ensure the library quality. The purified libraries were checked for the size by the Bioanalyzer 2100 (Agilent, St. Clara, CA, USA) using the DNA 1000 Lab Chip. The sequencing was performed through an Illumina sequencing platform (HiSeqTM 2500) by taking a library with an average size of >300 bp and Illumina HiSeq 151 × 2 PE read technology (Darshan et al., 2020).

### Transcriptome Data Analysis

#### Bioinformatics Analysis

The raw reads were checked for quality by FastQC (version 0.10.1, www.bioinformatics.babraham.ac.uk/projects/fastqc/). The reads with high-quality were mapped against the reference genome of *Lycopersicon esculentum*, accession number GCA_000188115 using Tophat (version: 2.1.0) (https://ccb.jhu.edu/software/tophat/index.shtml).

#### Differential Analysis

The gene expression was estimated for the assembled reads and cufflinks program module (http://cole-trapnell-lab.github.io/cufflinks) was used to quantify the transcripts. The gene expression level was quantified by RNA-seq by expectation maximization (RSEM) tool available at https://deweylab.biostat.wisc.edu/ rsem/ in the form of fragments per kilobase of exon per million mapped reads (FPKM) (Li and Dewey, 2011). The number of reads mapped to unigenes for each sample was calculated for each sample using SAMtools (version 0.1.19). Later, the differential analysis was carried out by using Cuffdiff with filters set as log2FC and *p*-values of 0.05. The heat map was prepared by using R package, such as Cummerbund (Goff et al., 2012), and the volcano plots were drawn by using ggplot2 (Wickham, 2016) with parameters set at default (Darshan et al., 2020).

The individual and combined unigenes of samples were functionally annotated by aligning to the non-redundant (NR) protein database (version 36) employing BLASTX version 2.2.31 (Suzuki et al., 2015) with E-value of 1 × e^−3^. Later, the functional annotation of the assembled contigs was performed by a Blast2GO software V 3.0 (https://www.blast2go.com) (Conesa et al., 2005). Further, the predicted proteins were mapped for the involvement in biochemical pathways by subjecting those to pathway analysis using the Kyoto Encyclopedia of Genes and Genomes (KEGG) (Ogata et al., 1999) database.

#### Gene Expression Analysis by qRT-PCR

The Illumina sequencing was further validated by using qRT-PCR. For this, 12 candidate genes related to induced resistance signaling pathways, such as SA, JA, ethylene, and phenylpropanoid pathways and genes exclusively expressed in biocontrol treated tomato plants were selected. All the qRT-PCR experiments were conducted for five time points (6, 12, 24, 48, and 96 hpi) after Cg-2 treatment with 0 hpi as control and each sample with six replicates (three biological replicates × two technical replicates). The RNA was extracted by TRIzol method and cDNA was synthesized by using the Thermo Fisher Scientific Verso cDNA Synthesis Kit as mentioned above.

The qRT-PCR reaction mix was prepared using the specific primer pairs to tomato genes as previously specified (Supplementary Table 2), and *SlEF* (Elongation factor) was used as internal control (Rotenberg et al., 2006). The dissociation curve of each gene determines the specificity of the primers. The relative fold change in gene expressions was calculated by using the 2^−ΔΔCt^ method (Kenneth and Thomas, 2001).

## Results

### Plant Growth

**Figure 1:**
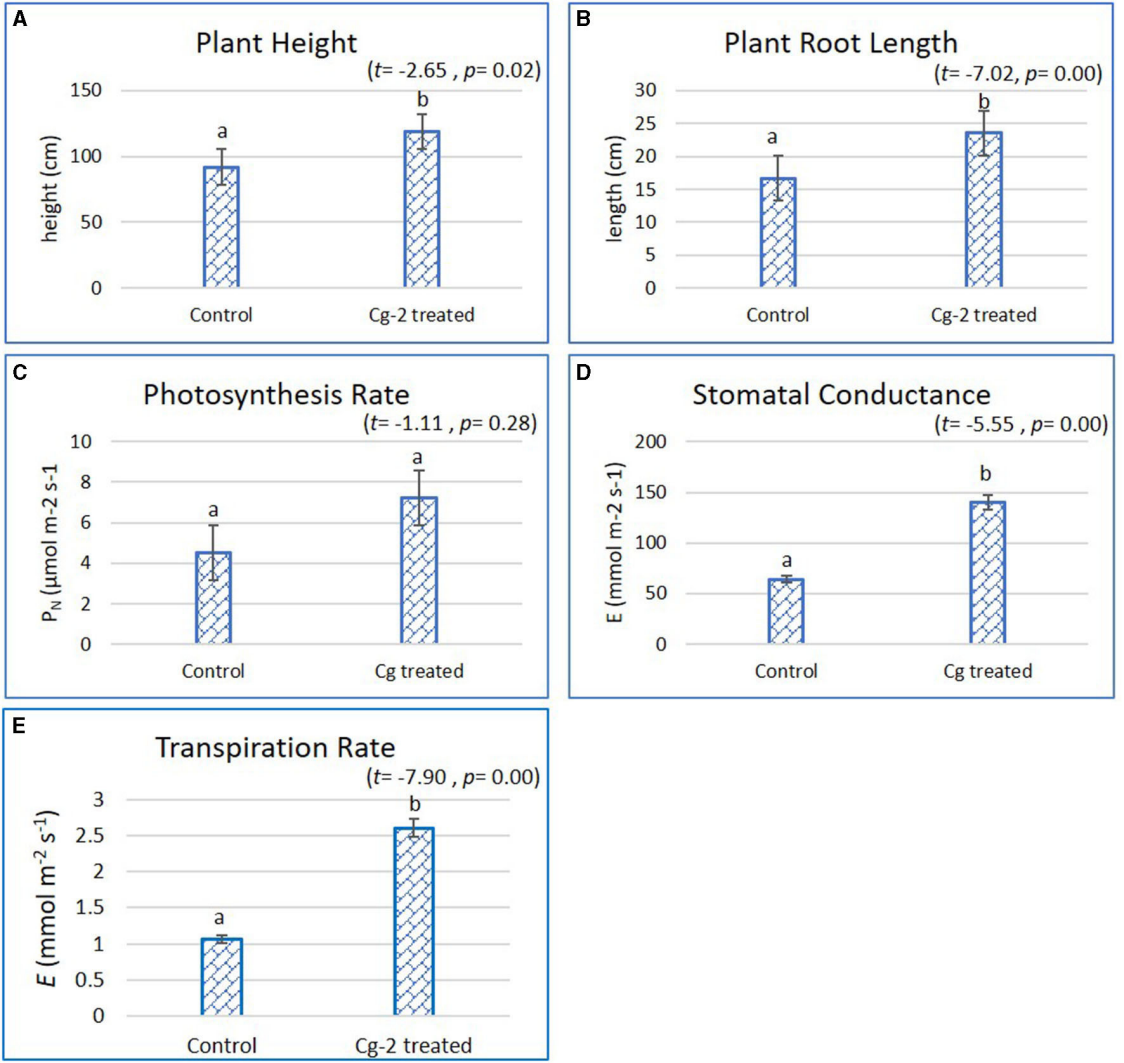
Effect of Chaetomium globosum (Cg-2) on growth and development of tomato plants (cv. Pusa Rohini). **(A)** Plant Height; **(B)** Plant Root Length; **(C)** Photosynthesis Rate; **(D)** Stomatal Conductance and **(E)** Transpiration Rate.

The plant growth parameters were statistically significantly different when treated with biocontrol agent Cg-2 and were analyzed by Student's *t*-test with SPSS (version 27.0). The biocontrol treated plants had better plant growth which was evident from increase in plant height by 26.7 cm, i.e., the mean plant height of 118.50 cm for biocontrol treated plants and 91.8 cm for untreated plants (*t* = −2.65, *p* = 0.02, α = 0.05, i.e., *p* < α) (Figure 1A). The plant root length increased by 6.9 cm from 16.6 cm for untreated to 23.5 cm for treated plants (*t* = −7.02, *p* = 0.00, α = 0.05, i.e., *p*< α) (Figure 1B). The comparative results for stomatal conductance (*t* = −5.55, *p* = 0.00, α = 0.05, i.e., *p* < α) and transpiration rate (*t* = −7.90, *p* = 0.00, α = 0.05, i.e., *p* < α) for biocontrol treated vs. untreated plants showed statistically significant difference, whereas the photosynthesis rate did not show statistically significant difference (*t* = −1.11, *p* = 0.28, α = 0.05, i.e., *p*> α). The photosynthesis rate (*P*_*N*_) was recorded 7.4 and 4.7 μmol m^−2^ s^−1^ for Cg-2 treated and untreated plants, respectively (Figure 1C). At the same time, stomatal conductance (*gs*) was recorded as 133.32 mmol m^−2^ s^−1^ for Cg-2 treated and 66.27 mmol m^−2^ s^−1^ for control plants, i.e., two times enhancement in *gs* on biocontrol treatment (Figure 1D). The transpiration rate (*E*) was enhanced by 1.54 mmol m^−2^ s^−1^ with biocontrol treatment, which was documented as 2.6 and 1.06 mmol m^−2^ s^−1^ for biocontrol treated and control plants (Figure 1E). Overall, Cg-2 treated plants performed better on assessment for various morphological and physiological parameters of growth and development (Figure 1).

### *C. globosum* Induced Systemic Resistance in Tomato

**Figure 2:**
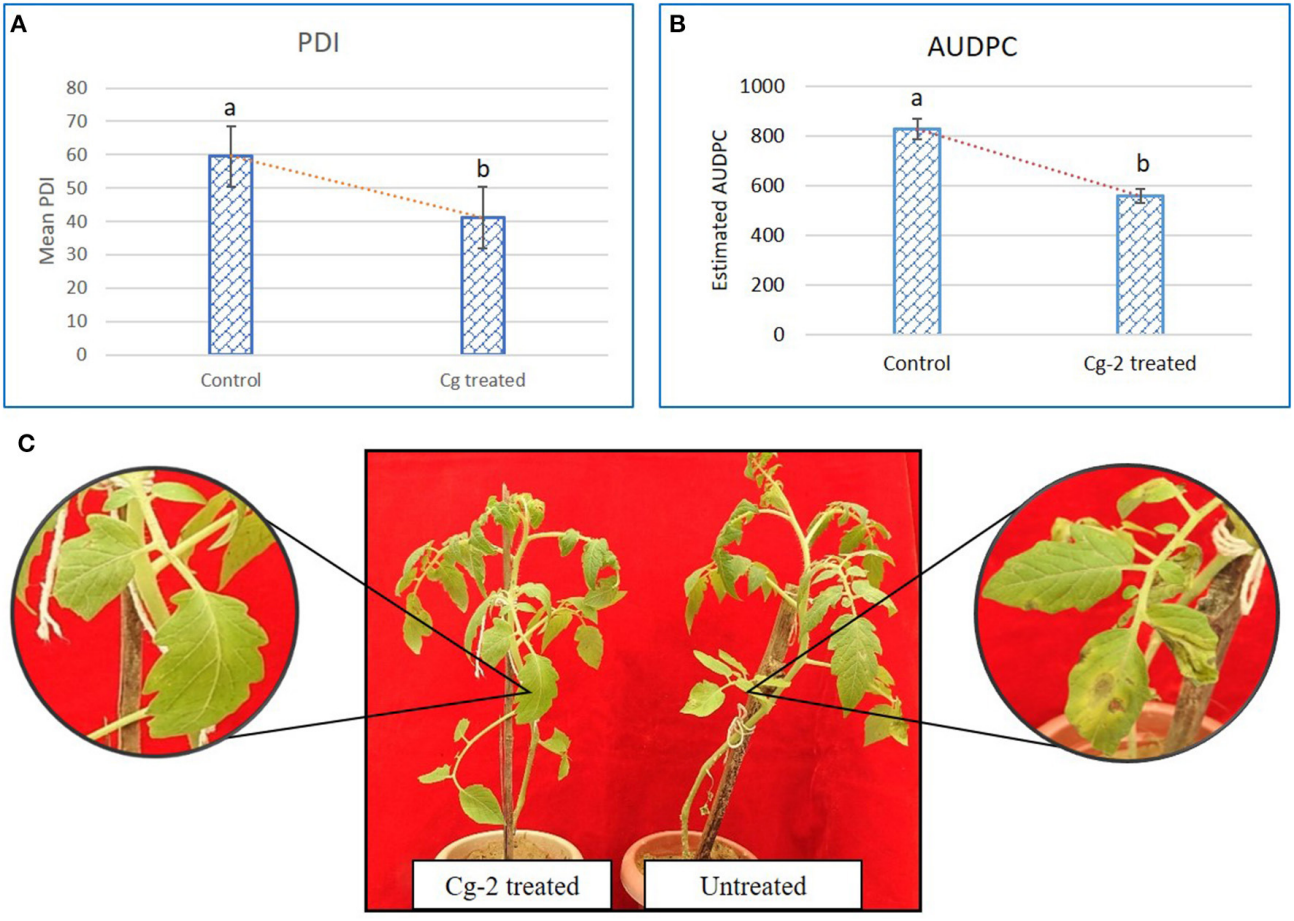
*Chaetomium globosum* induced systemic resistance against early blight in tomato plants. **(A)** Mean PDI of control plant and Cg-2 treated plants. **(B)** Area under disease progress curve by plotting disease progress curve with PDI at *Y*-axis and time points 0, 7, 14, and 21 dpAi at *X*-axis. **(C)** *A. solani* symptom development in *C. globosum* treated and untreated control plant.

The mean PDI was 59.44 for control plants (untreated plants) and 41.08 for Cg-2 treated plants (Figure 2A) and results showed 30.88% disease reduction in early blight disease due to Cg-2 induced resistance in tomato (Figure 2C). The AUDPC was 828.31 for control plants whereas, it was 559.82 for Cg-2 treated plants (Figure 2B; Table 1).

**Table 1:** The plant disease index (PDI) and the area under disease progress curve (AUDPC) for untreated and treated plants.

	**7dpAi**	**14dpAi**	**21dpAi**	**PDI mean**	**AUDPC**
Control	50.00	58.33	70.00	59.44	828.31
(water + *A. solani*)					
Cg-2 treated	33.33	36.66	53.3	41.09	559.82
(Cg-2+ *A. solani*)					

*The data are means of eight replicates (Student's t-test, P < 0.05) dpAi—days post A. solani inoculation*.

### Gene Expression Analysis of Marker Genes of Hormone Signaling Pathways Using qRT-PCR

**Figure 3:**
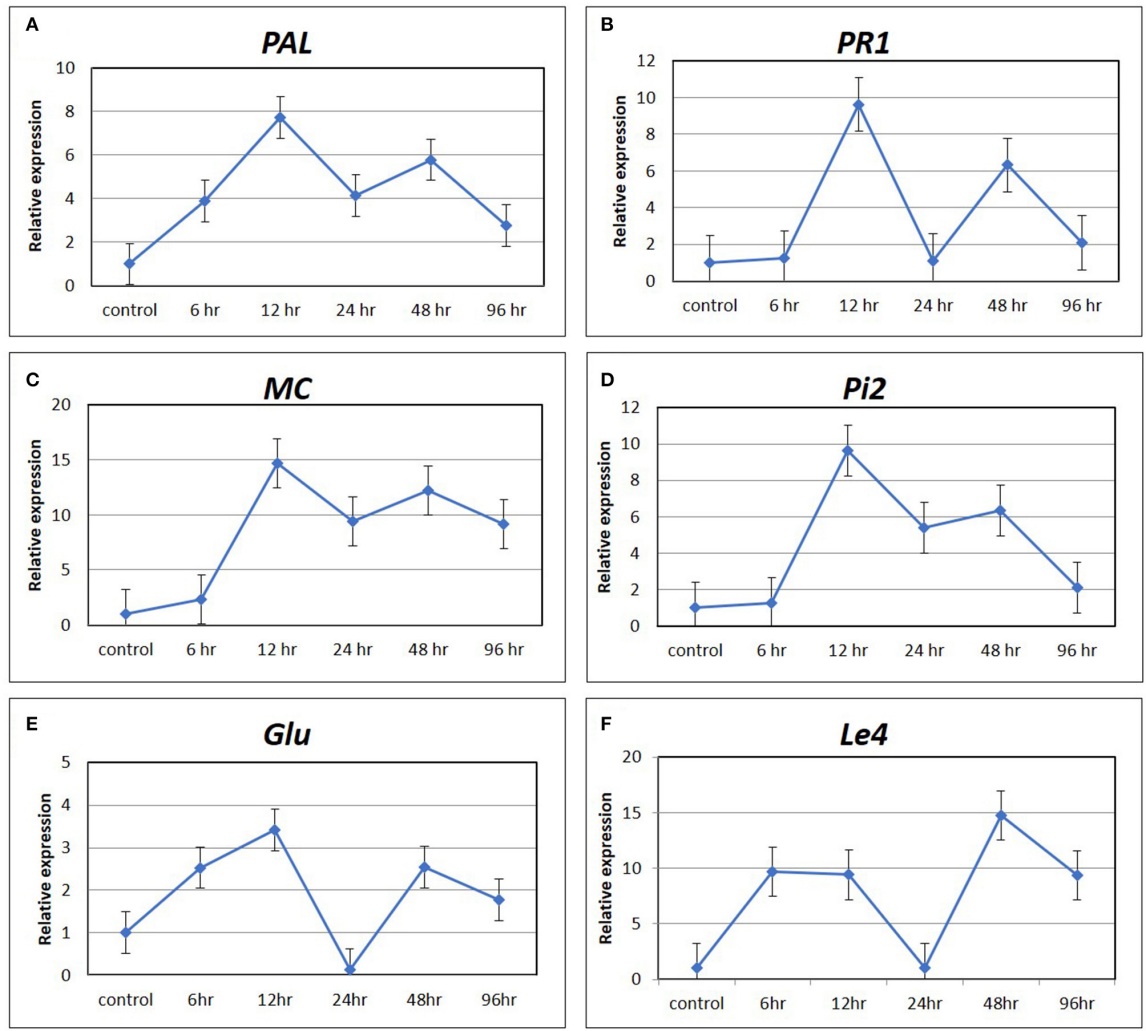
The expression pattern of marker gene of defense signaling hormone transduction pathways by qRT-PCR **(A)** *PAL and* **(B)** *PR1* of SA mediated signaling; **(C)** *MC* and **(D)** *Pi2* of JA signaling; **(E)** *Glu* of ET mediated signaling; and **(F)** *Le4* of ABA signaling.

The expression pattern of six marker genes of hormone signaling pathways, such as *PAL* and *PR1* for SA pathway, *MC* and *PiII* for JA pathway, *Glu* for ET pathway, and *Le4* for ABA pathway at five time points (6, 12, 24, 48, and 96 hpCi) after Cg-2 treatment as compared with control (0 hpCi) revealed maximum expression of most of the genes at 12 hpCi. The *PAL* and *PR1* genes showed maximum fold change at 12 hpCi, i.e., upto ~eight-fold and ~ten-fold, respectively, followed by 48 hpCi exhibiting six-fold upregulation for both the genes. The marker genes of JA pathway *MC* and *Pi2* were expressed maximum at 12 hpCi as ~15- and ~ten-fold, respectively. The *Glu* gene of ET signaling shows ~3.5-fold and ~2.5-fold upregulation at 12 and 48 hpCi in Cg-2 treated plants. In the same trend, *Le4* gene of ABA pathway was expressed up to maximum extent, i.e., fifteen-fold at 6 hpCi. Overall, most of the marker or signature genes of defense hormone signal transduction pathways showed a maximum expression at 12 hp root inoculation with Cg-2. The time point with the highest level of gene expression can be easily visualized by observing peak at 12 phCi in line graphs with relative fold change on *Y*-axis and time points on *X*-axis (Figure 3).

### RNA-Seq Data Statistics

RNA-seq data yielded an average of 19–20 million reads by using the Illumina HiSeq 2000 mRNA sequencing platform with an average read length of 2,000 bp (Supplementary Figure 1). The mapping percentage with reference genome of *S. lycopersicum* ranged from 84 to 90%. The transcriptome assembly statistics of the four processed samples are given in Supplementary Table 3. The density plot was generated to present the distribution of RNA seq read count (FPKM) of Cg-2 treated and untreated plants (Supplementary Figure 2). Out of the total quality-filtered reads, reads which had a Phred score value ≥ Q30, indicated that RNA-seq data are reliable and of good quality and can be used for further analysis. The statistics of Illumina sequencing after trimming is presented as Supplementary Table 3. The RNA-seq data were deposited at the NCBI database under bio-project number—PRJNA721530 with bio-sample accessions number: SAMN18719853, SAMN18719854, SAMN18719855, and SAMN18719856. The scatter plot was generated using CummeRbund from gene expression data (FPKM value) of Cg-2 treated and untreated plants. The genes with similar expressions are distributed near the diagonal, whereas outliers are visualized as dots away from the diagonal line Supplementary Figure 3.

### Functional Analysis of DEGs Induced by Cg-2 at 12 hpCi

**Figure 4:**
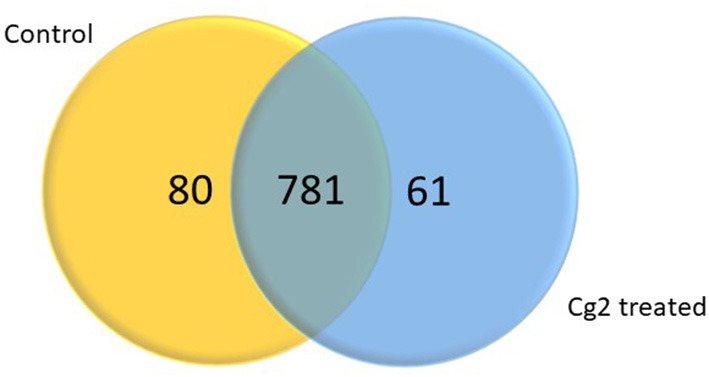
Venn diagram showing the differentially expressed genes (DEGs) significantly expressed exclusively in control plant, Cg-2 treated plant and mutually expressed in both conditions. (Fold change <-2 or >2 and *P* < 0.05).

**Figure 5:**
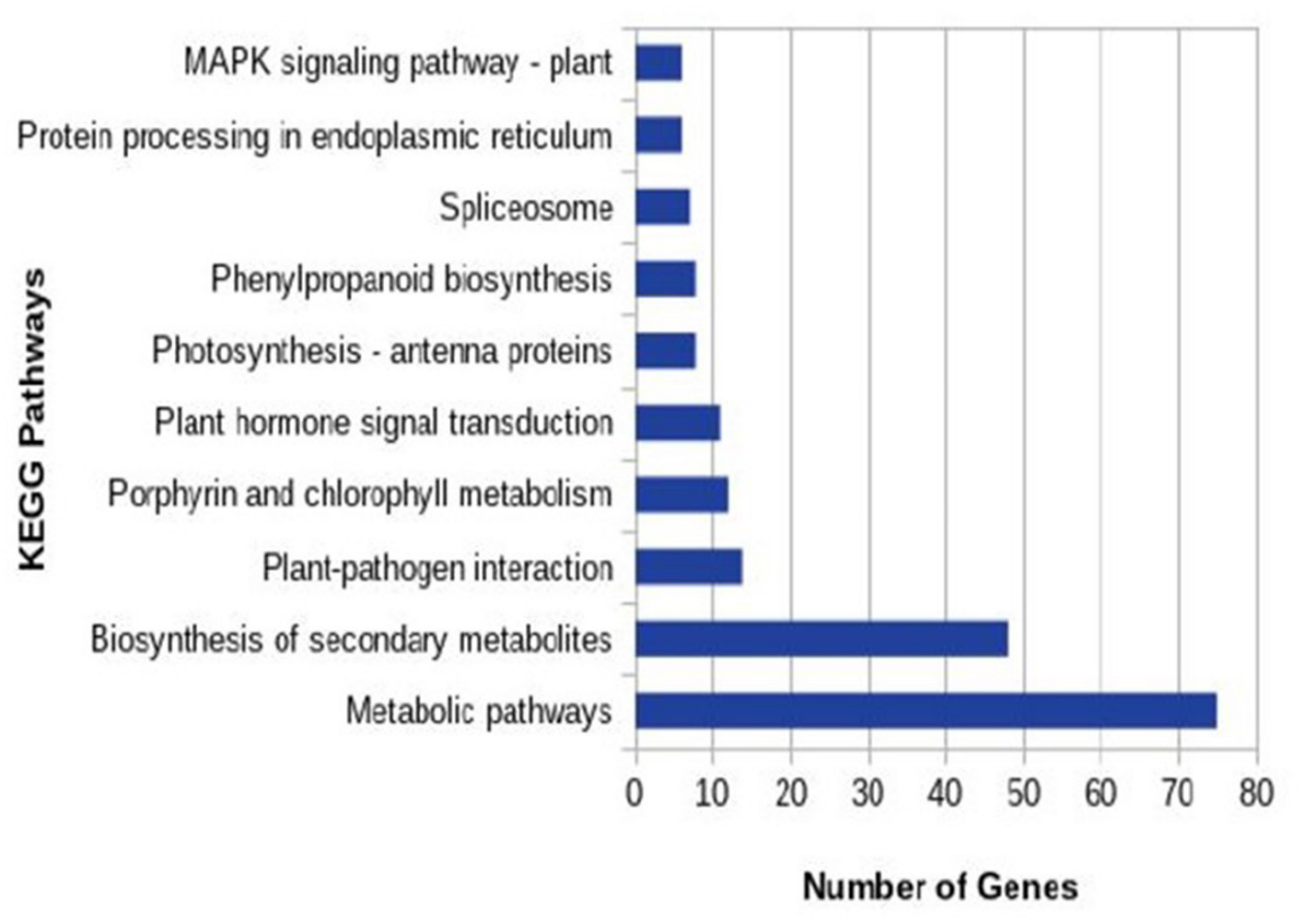
Column graph representing the enriched Kyoto Encyclopedia of Genes and Genomes (KEGG) pathways based on the upregulation of DEGs in Cg-2 treated plants in comparison to untreated (control).

**Figure 6:**
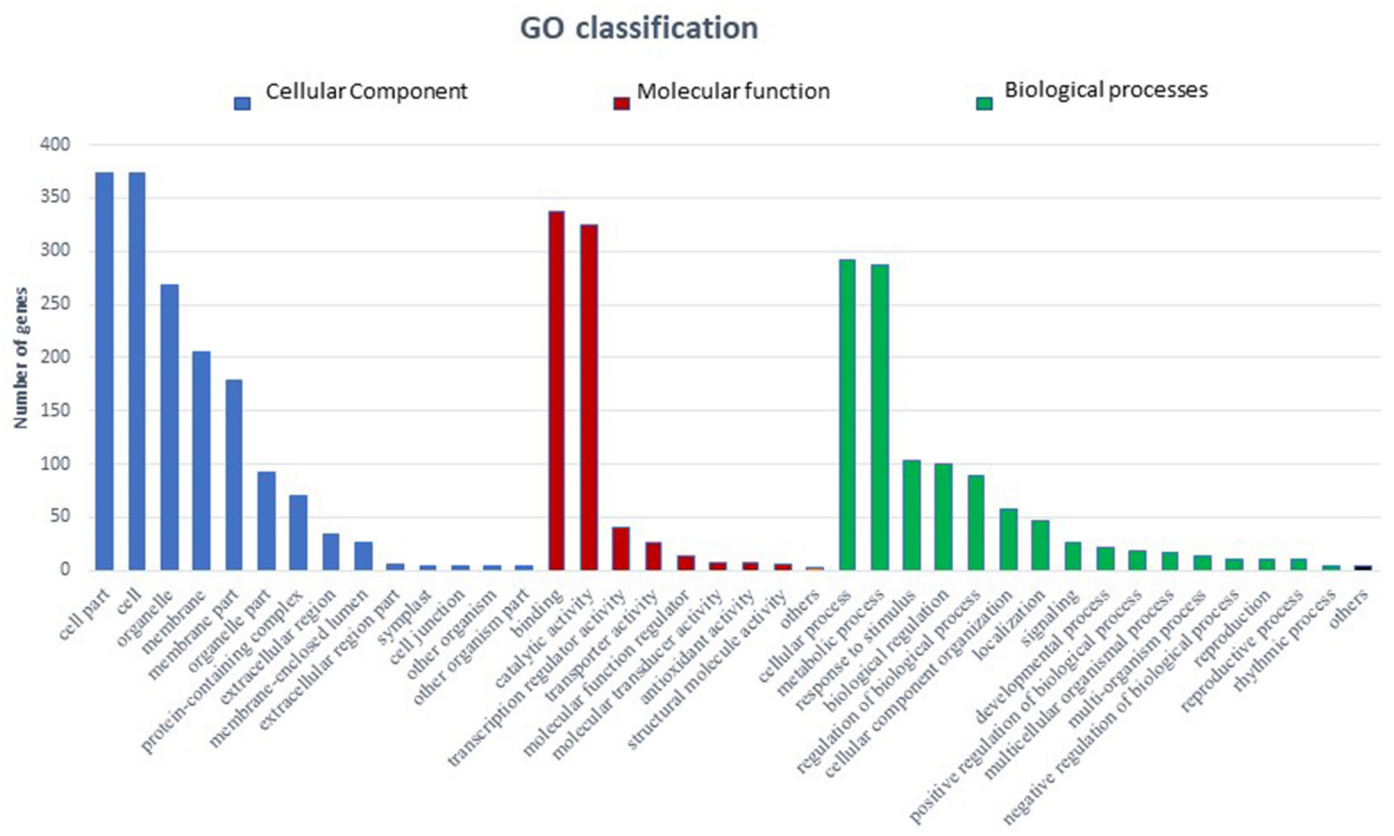
Bar graph depicting the number of upregulated DEGs belonging to different Gene Ontology (GO) categories in tomato by GO enrichment analysis. (Blue bars represents cellular component category, red bars represents molecular function category, and green bars represents biological function category).

**Figure 7:**
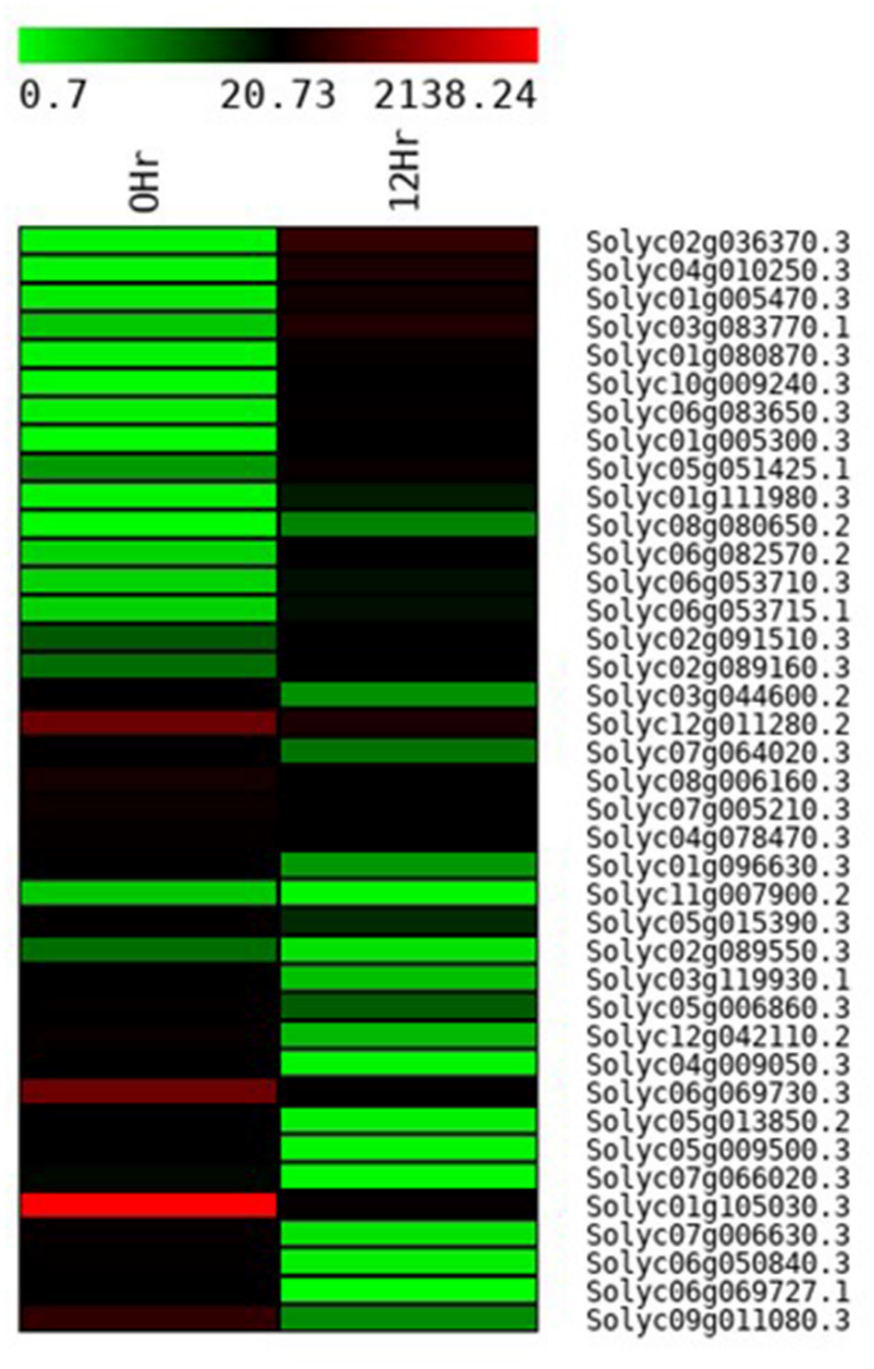
Heat map displaying the change in the expression pattern of genes in tomato plant on the treatment with Cg-2 biocontrol agent. (Green to black to red marks the increase in the expression of gene).

In total 22,473 specific DEGs were expressed in tomato at 12 h after Cg-2 inoculation as compared with control plants without Cg-2 treatment and among these 922 DEGs had fold change (−2) to (+2) with *p*<0.05 (Supplementary Table 4). The significant genes above the threshold of FDR and log FC are presented as red dots in the volcano plot (Supplementary Figure 5). Out of 922 DEGs, 61 DEGs were expressed exclusively in control plants (0 hpCi), 80 DEGs at 12 hpCi and 781 DEGs were commonly expressed at both time points 0 and 12 hpCi (Figure 4; Supplementary Figure 4). The top 50 DEGs upregulated in tomato plants in response to *C. globosum* Cg-2 treatment are presented in Supplementary Table 4. The KEGG pathway analysis revealed that most of the DEGs belonged to 10 KEGG pathways with maximum DEGs (i.e., 1,370 DEGs) related to “metabolic pathways,” “biosynthesis of secondary metabolites” (762), “ribosome” (219), “carbon metabolism” (167), “plant hormone signal transduction” (155), “biosynthesis of amino acids” (145), “plant-pathogen interaction” (135), “protein processing in endoplasmic reticulum” (124), “phenylpropanoid biosynthesis” (105), and “MAPK signaling pathway” (103) in plant (Figure 5 and Supplementary Table 6). Gene Ontology (GO) classification indicated 1,647 DEGs with 15 GO terms belonging to the cellular component category, 756 DEGs with 10 GO terms belonged to molecular function category, and 1,113 DEGs with 20 terms belonged to the biological processes (Supplementary Table 7). The maximum DEGs belonged to binding (GO:0005488) 338 DEGs, catalytic activity (GO:0003824) 324 DEGs, metabolic process (GO:0008152) 287 DEGS, cellular process (GO:0009987) 292 DEGs), response to stimulus (GO:0050896) 103 DEGs, biological regulation (GO:0065007) 100 DEGs, and transcription regulator activity (GO:0140110) 41 DEGs (Figure 6). The heat map depicted the important genes of metabolic processes, secondary metabolites biosynthesis, signaling pathways, and some uncharacterized genes which are highly upregulated or downregulated in tomato plants on Cg-2 inoculation (Figure 7; Table 2).

**Table 2:** The gene IDs, protein names, and fold change of DEGs in tomato plants treated with Cg-2 are represented in the heat map.

**Gene_id**	**Gene**	**Protein Name**	**Fold change**
XLOC_013553	Solyc02g036370.3	HTH myb-type domain-containing protein	8.28
XLOC_018512	Solyc04g010250.3	Hydrolase_4 domain-containing protein	7.57
XLOC_002084	Solyc01g005470.3	Uncharacterized protein	6.31
XLOC_015760	Solyc03g083770.1	PMEI domain-containing protein	5.86
XLOC_003133	Solyc01g080870.3	Uncharacterized protein	5.70
XLOC_005703	Solyc10g009240.3	3-ketoacyl-CoA synthase (EC 2.3.1.-)	5.45
XLOC_026380	Solyc06g083650.3	Lipase_GDSL domain-containing protein	5.38
XLOC_000015	Solyc01g005300.3	PAS domain-containing protein	5.37
XLOC_023274	Solyc05g051425.1	FSH1 domain-containing protein	3.53
XLOC_004213	Solyc01g111980.3	Aa_trans domain-containing protein	3.51
XLOC_030054	Solyc08g080650.2	Uncharacterized protein	3.51
XLOC_026316	Solyc06g082570.2	NAB domain-containing protein	3.50
XLOC_025568	Solyc06g053710.3	Ethylene receptor	2.20
XLOC_025568	Solyc06g053715.1	Uncharacterized protein	2.20
XLOC_014804	Solyc02g091510.3	Uncharacterized protein	1.53
XLOC_012971	Solyc02g089160.3	Cytochrome P450 85A1 (C6-oxidase) (Dwarf protein)	1.53
XLOC_015326	Solyc03g044600.2	Uncharacterized protein	−1.93
XLOC_010635	Solyc12g011280.2	Chlorophyll a-b binding protein, chloroplastic	−1.93
XLOC_027563	Solyc07g064020.3	SHSP domain-containing protein	−1.95
XLOC_030251	Solyc08g006160.3	Uncharacterized protein	−1.97
XLOC_027695	Solyc07g005210.3	Temperature-induced lipocalin	−1.97
XLOC_020808	Solyc04g078470.3	CycD3;3 protein	−1.98
XLOC_001340	Solyc01g096630.3	ACI112	−2.00
XLOC_006942	Solyc11g007900.2	Uncharacterized protein	−2.00
XLOC_021477	Solyc05g015390.3	Uncharacterized protein	−2.01
XLOC_014699	Solyc02g089550.3	Protein kinase domain-containing protein	−2.01
XLOC_018145	Solyc03g119930.1	Uncharacterized protein	−2.01
XLOC_022341	Solyc05g006860.3	Thioredoxin domain-containing protein	−2.02
XLOC_009906	Solyc12g042110.2	Uncharacterized protein	−3.94
XLOC_019837	Solyc04g009050.3	Uncharacterized protein	−3.98
XLOC_024544	Solyc06g069730.3	Chlorophyll a–b binding protein, chloroplastic	−4.15
XLOC_022651	Solyc05g013850.2	Uncharacterized protein	−4.24
XLOC_022466	Solyc05g009500.3	Uncharacterized protein	−4.26
XLOC_027660	Solyc07g066020.3	Uncharacterized protein	−4.26
XLOC_001713	Solyc01g105030.3	Chlorophyll a-b binding protein CP24 10A, chloroplastic	−4.8
XLOC_027762	Solyc07g006630.3	Uncharacterized protein	−4.9
XLOC_024041	Solyc06g050840.3	Uncharacterized protein	−4.96
XLOC_024543	Solyc06g069727.1	Uncharacterized protein	−5.04
XLOC_032896	Solyc09g011080.3	ATPase_AAA_core domain-containing protein	−5.46

### Analysis of DEGs Associated With Plant Hormone Signaling Pathways

**Figure 8:**
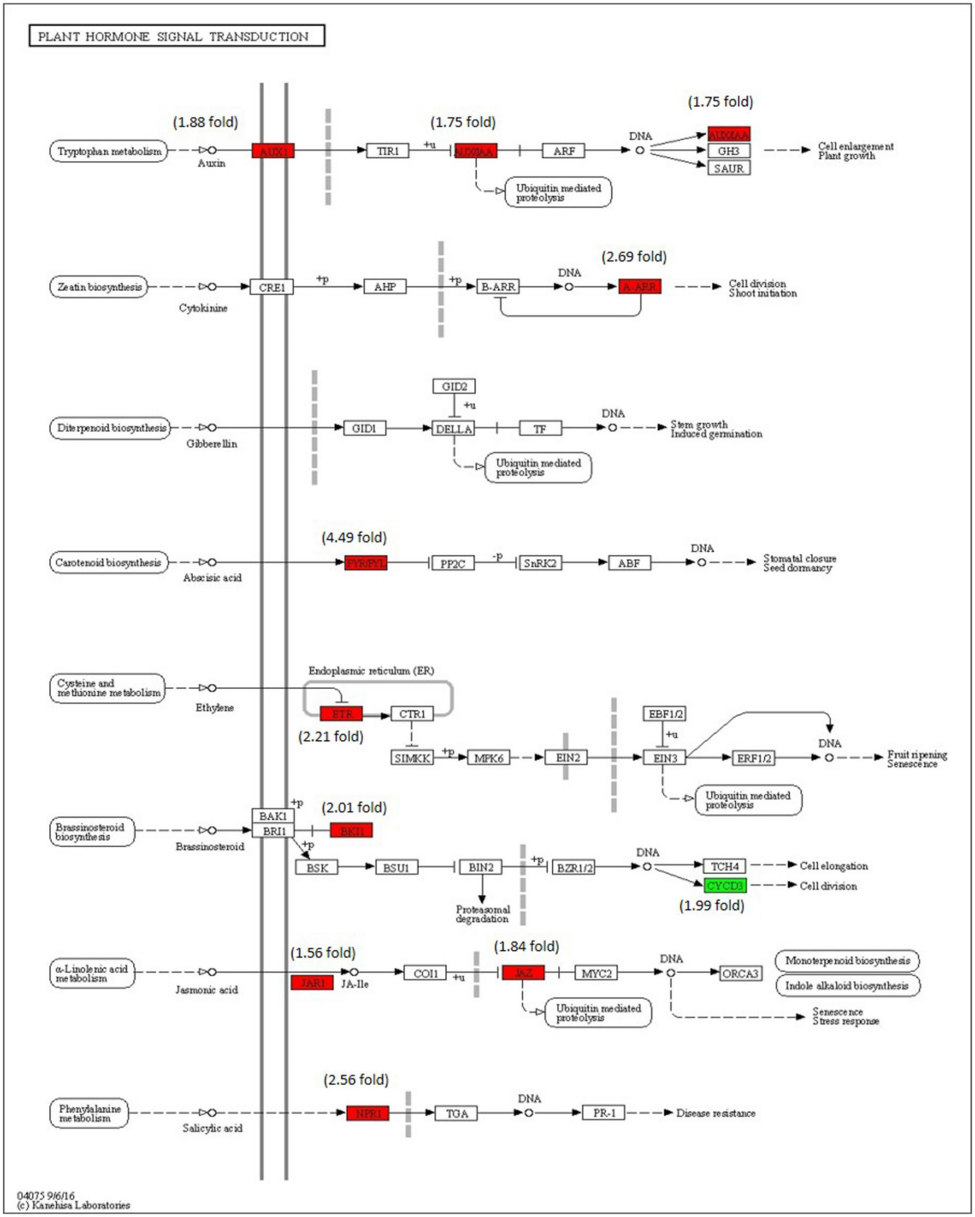
Modulation of gene expression in plant hormone signaling pathways (salicylic acid (SA), jasmonic acid (JA), ethylene (ET) and brassinosteroid pathways) in tomato plant by Cg-2 treatment. (Red color represents upregulated genes and green color represents downregulated genes).

The enrichment of phytohormone signaling transduction pathways is visualized in Figure 8. The red box indicates the upregulation of genes and the green box marks the downregulation of genes, which allows the depiction of the involvement of the hormone signal transduction pathway. The gene JAR1 (Solyc10g011660) (1.56-fold) and JAZ (Solyc01g005440) (1.84-fold) participating in JA signal transduction and nonexpresser of pathogenesis-related genes 1 (NPR1) (Solyc07g040690) (2.56-fold), a key regulator of SA signaling are upregulated. The ETR (Solyc12g011330) (2.21-fold) gene of ET signal transduction which negatively regulated ET signaling is upregulated and PYR/PYL (Solyc08g076960) (4.49-fold) that positively transduced the ABA signaling are also upregulated. The absence of participation of the brassinosteroid pathway is marked by the downregulation of CYCD3 (Solyc01g080190) and upregulation BKI1 (Solyc04g011520), negative regulator of brass inosteriod signaling.

The phytohormones involved in the plant growth and development are auxin, cytokinin, and gibberellic acid. The AUX1 and indole-3-acetic acid (IAA) of auxin pathway are upregulated along with type-A arabidopsis response regulator (A-ARR) of cytokinin signal transduction, whereas gibberellic acid signaling remained in an innate mode without any modulation in gene expression.

### Expression Analysis of Key Genes Engaged in Phenylpropanoid Biosynthesis

**Figure 9:**
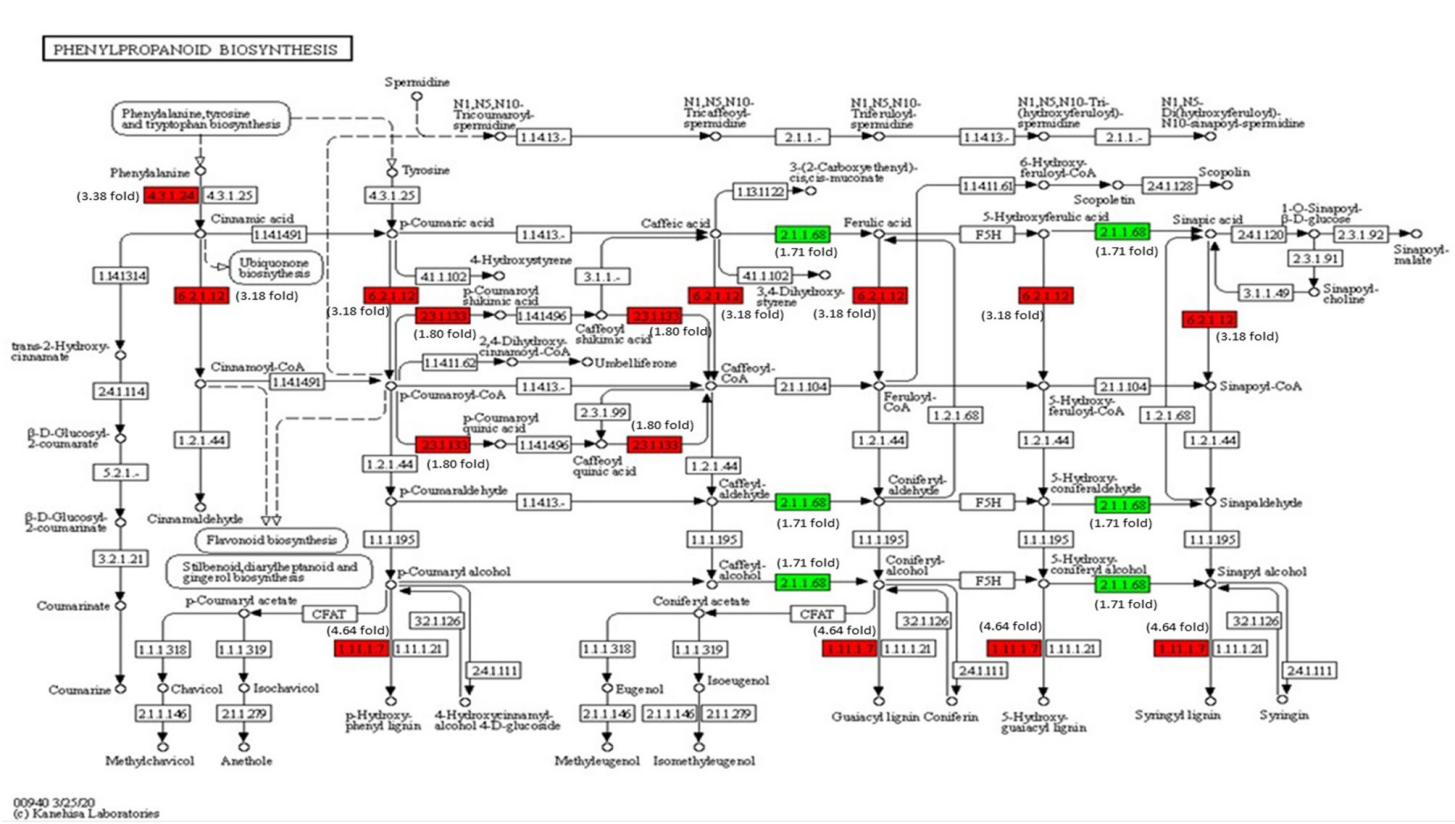
Modulation of gene expression in phenylpropanoid biosynthesis in tomato plant by Cg-2 treatment. (Red color represents upregulated genes, and green color represents downregulated genes).

The transcriptome analysis reveals that phenylalanine ammonia-lyase (*PAL*) (Solyc09g007920), cinnamic acid 4-hydroxylase (*C4H*) (Solyc12g011330), and 4-coumarate-CoA ligase (*4CL*) (Solyc05g047530) genes of phenylpropanoid biosynthesis pathway were significantly upregulated on inoculation with Cg-2 (Figure 9). The key genes of lignin formation, such as p-coumarate 3-hydroxylase (*C3H*) (Solyc01g096670) and cinnamoyl-CoA reductase (*CCR*) (Solyc08g076790) were significantly elevated in tomato plants treated with Cg-2. The peroxidase (*POX*) (Solyc02g079500), which is responsible for lignin polymerization, is also significantly upregulated in biocontrol treated plants. The lignin formation is important to restrict the subsequent infection by *A. solani* in biocontrol treated plants.

### Modulation of Gene Expression in MAPK Signaling Pathway

**Figure 10:**
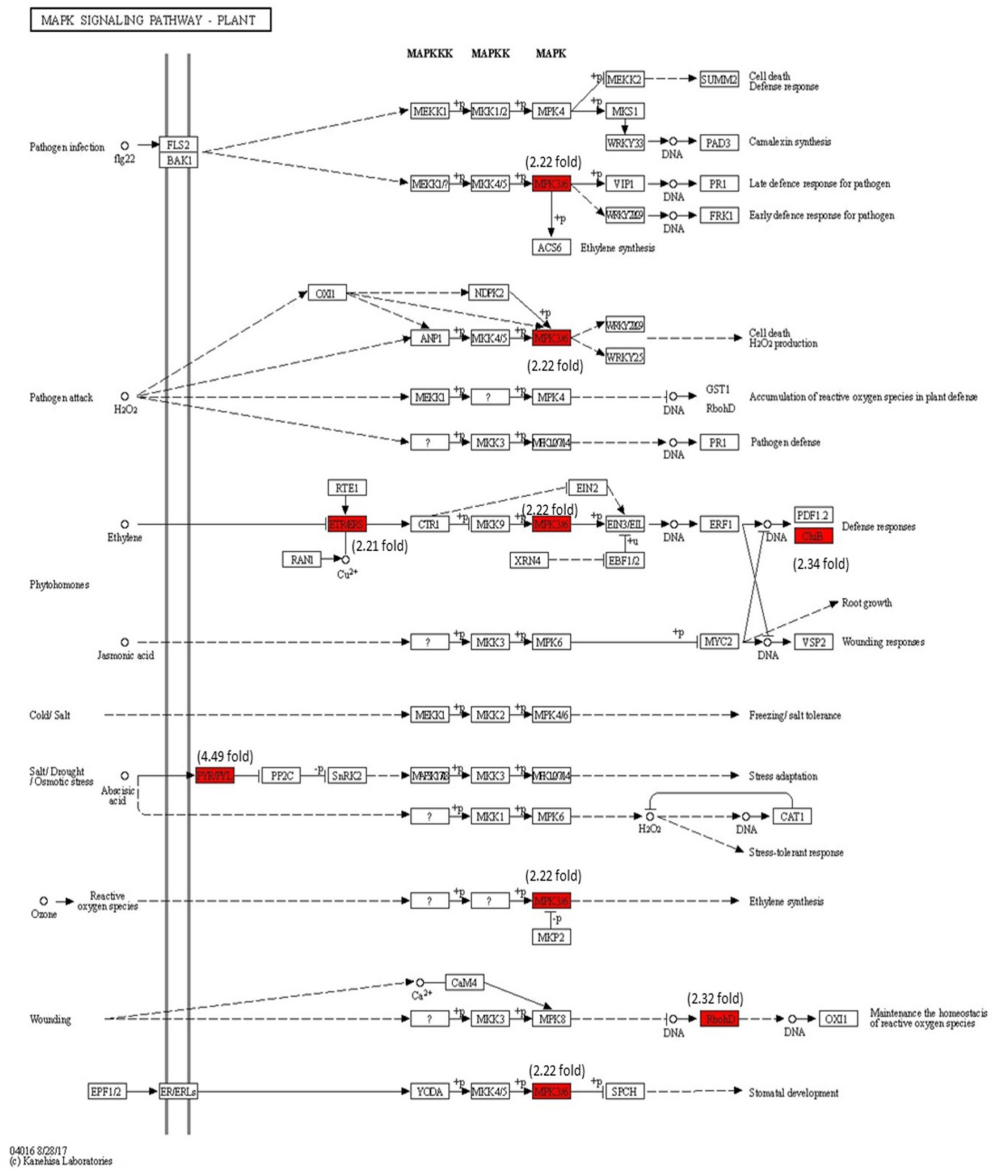
Modulation of gene expression in MPK signaling transduction pathway in tomato plant by Cg-2 treatment. (Red color represents the upregulated genes, and green color represents the downregulated genes).

The modulation of gene expression in MAPK cascades following the signal transduction during pathogen attack, JA signaling, ET signaling, and reactive oxygen species (ROS) are marked in Figure 10. The MPK3/6 (Solyc06g005170) (2.22-fold) involved in downstream signaling in H_2_O_2_, ET, SA, and ROS are significantly upregulated with Cg-2 inoculation as compared with mock-inoculated control plant. The NADPH kinase, RbohD (2.32-fold), which is involved in phosphorylation during defense signaling for plant immunity, is upregulated in biocontrol treated plants.

### Validation of Transcriptomic Data by qRT-PCR Analysis

**Figure 11:**
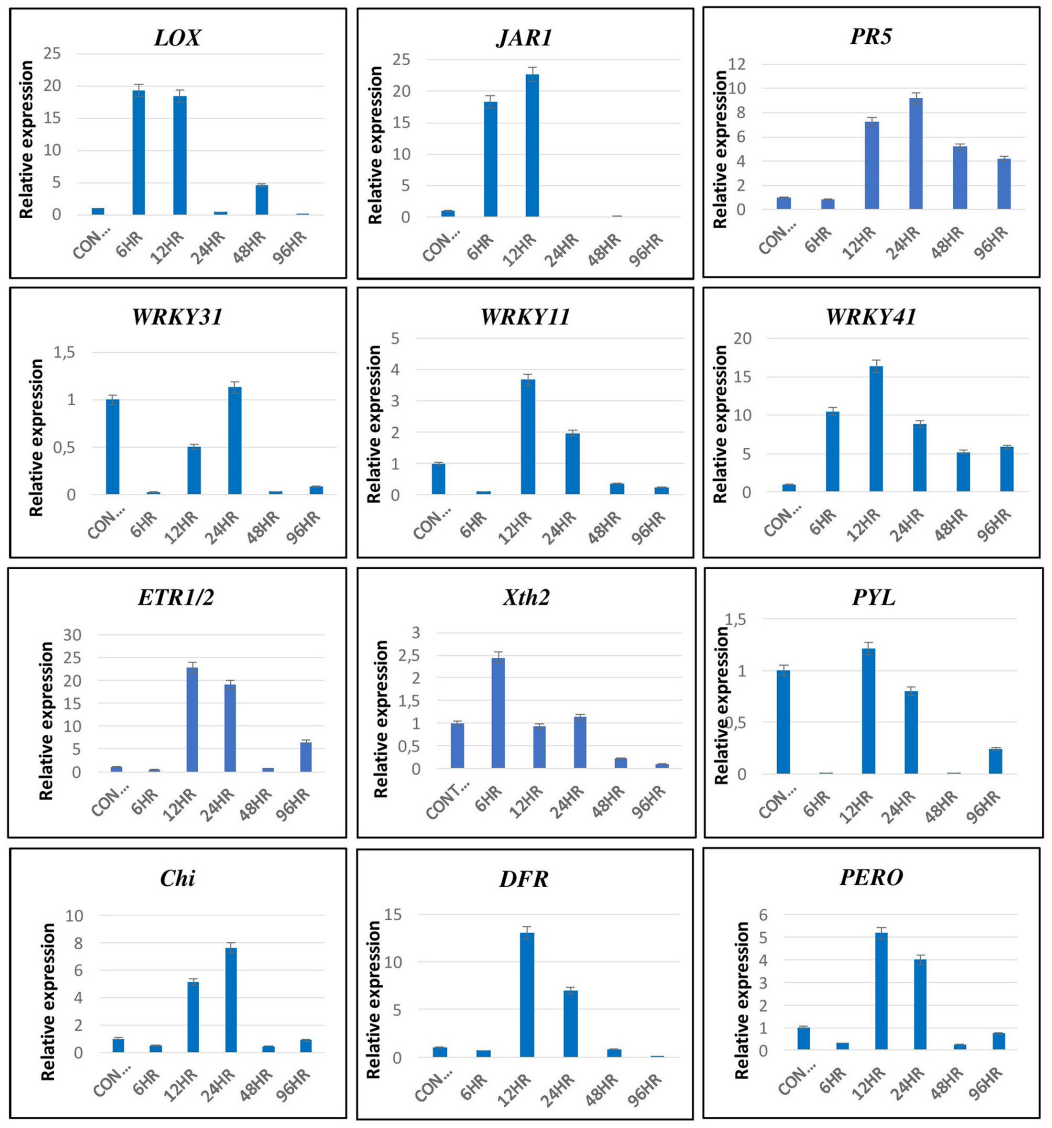
The validation of expression of selected genes by qRT-PCR showed significant difference in their expression at different time intervals. Error bars show ± SD among the biological triplicates.

The transcriptomics data were validated by qRT-PCR using primers of genes related to various signaling pathways, such as *LOX* & *JAR1* related to JA signaling pathways; *PR5, WRKY11, WRKY31*, and *WRKY41* related to SA signaling pathway; *ERF1/2* & *Xth2* related to ET signaling pathway; *PYL* related to ABA; and chitinase (*Chi*), *DFR* and peroxidase (*PERO*) related to the defense responses of the plant (Figure 11). The expression pattern of these genes by qRT-PCR was in correlation with that observed in the transcriptomic data. The *LOX* and *JAR1* were expressed maximum at 6 and 12 hpCi. The *PR1* and *WRKY 41* are expressed throughout from 6 to 96 hpCi with maximum at 24 and 12 hpCi, respectively. The other two WRKY genes *WRKY31* and *WRKY11* showed the highest expression at 12 and 24 hpCi. Among the genes of ET pathway, *ERF1/2* was highly expressed at 12 and 24 hpCi, whereas Xth2 was expressed at 6, 12, and 24 hpCi. The *PYL* of the ABA pathway did not show significant fold change at different time points. The genes related to defense, such as *Chi, DFR*, and *PERO* showed high expression on 12 and 24 hpCi.

## Discussion

*Chaetomium globosum* is a biocontrol agent which has been found effective against a large number of plant pathogens operating through various mechanisms, such as mycoparasitism, antibiosis, nutrient competitiveness, and inducing systemic resistance in plants (Soytong et al., 2001; Sandhu et al., 2021a). The induced resistance mechanism of *C. globosum* is least explored except a few reports stating the effectiveness of induced resistance in wheat against tan spot and spot blotch diseases (Istifadah and McGee, 2006; Biswas et al., 2012). This study demonstrated that inoculation of C. *globosum* to tomato roots enhanced the defense response in leaves against foliar pathogen, *A. solani* as characterized by inhibition of early blight disease by 30% that was observed in this study. Once induced defense was established in *C. globosum* treated plants, its molecular mechanism was explored by transcriptome analysis of tomato plants treated with *C. globosum* vs. untreated. In the RNA seq experiment, the counter inoculation by *A. solani* was avoided to get true to type information about the transcriptome programming in tomato exclusively by *C. globosum*. However, this study is the first effort to explore the molecular mechanism of systemic resistance induced by *C. globosum* in plants to enhance the defense responses against phytopathogen. Most commonly systemic resistance induced in plants is either SAR or ISR. SAR is activated by plant pathogen through SA mediated signaling, whereas, ISR is activated by PGPRs or beneficial fungus, such as *Trichoderma* spp. through JA and ET signaling (Romera et al., 2019; Sandhu et al., 2021b,c). Most popular biocontrol fungal agent, *Trichoderma* spp. induce systemic resistance in plants which is commonly categorized as ISR mediated by JA/ET signaling pathway independent of SA. In contrast, some recent reports state that few species of *Trichoderma*, such as *T. longibrachiatum* H9 induce systemic resistance that is mediated by JA, ET, and SA signaling indicating the involvement of ISR and SAR simultaneously (Yuan et al., 2019). This marked the importance of this study to explore the induced systemic resistance in tomato by *C. globosum*. The temporal expression analysis of marker genes of hormone signaling pathways in tomato plants after root inoculation with potential strain Cg-2 revealed the maximum expression of genes at 12 hpCi. Therefore, the RNA seq was performed for control tomato plants (mock-inoculated with water) and biocontrol treated plants at 12 h post root inoculation with Cg-2. The analysis of transcriptomic data revealed the upregulation of genes related to biosynthesis of secondary metabolites, plant hormone signal transduction, biosynthesis of amino acids, plant-pathogen interaction, protein processing in endoplasmic reticulum, phenylpropanoid biosynthesis, and MAPK signaling pathway in tomato plants after root inoculation with Cg-2. Further, the inspection of gene modulation in hormone signal transduction pathways provided deep insight to decipher the defense signaling networks.

Jasmonic acid primarily involved in ISR is well characterized by oxylipin and synthesized by oxygenation of unsaturated fatty acids through lipoxygenases (LOX9 and LOX13) (Andreou et al., 2009). The precursor of JA, 12-oxo-phytodienoic acid (12-OPDA) is synthesized in plastids by the involvement of LOX13, allene oxide cyclase (AOC), and allene oxide synthase (AOS). The precursor 12-OPDA is transformed to JA in peroxisome (Borrego and Kolomiets, 2016). Later, biologically active JA, JAIle is produced by conjugation of isoleucine to JA through JAR1. The downstream JA signaling is activated by the binding of JAIle to the receptor complex of the Coronatine Insensitive1 (COI1)-Jasmonate Zim Domain (JAZ) (Wang et al., 2020). The transcriptomic data analysis reports the upregulation of most of the genes of JA biosynthesis and downstream signaling by ET. The expression pattern of genes related to JA biosynthesis and signaling of genes, such as *LOX, JAR1, ASC, Pi2*, and *MC* by qRT-PCR and transcriptomics marks the increase in expression specifically at 6, 12, and 48 hpi up to twenty-fold. The increase in expression of these genes indicates that JA mediated signaling is actively contributing to the systemic defense induced by Cg-2 in tomato plants. The JA mediated signaling indicated the activation ISR in systemic defense in tomato. The gene modulation showed the upregulation of ETR, the inhibitor of ET mediated signal transduction. It indicated that among JA and ET signaling components of ISR, only JA mediated signaling is contributing to the maximum extent in Cg-2 induced defense, whereas both the pathways play a significant role in ISR induced defense. The upregulation of ET mediated genes was observed only up to 2.5-fold, whereas JA mediated genes showed upregulation up to twenty-fold. A recent study on *T. longibrachiatum* H9 mediated plant systemic resistance against *Botrytis cinerea* also showed similar results that ISR induced by *T. longibrachiatum* H9 is JA mediated without any influence of ET signaling (Yuan et al., 2019).

Systemic acquired resistance signaling in plants usually involves several metabolites, such as methyl ester of SA (MeSA), the diterpenoid dehydroabietinal (DA), a glycerol-3-phosphate (G3P)-dependent factor, azelaic acid (AzA), and pipecolic acid (Pip). SA is synthesized from chorismate by two pathways: isochorismate synthase (ICS) and phenylalanine ammonia-lyase (PAL). In the ICS pathway, chorismate is first converted to isochorismate (IC) by an enzyme, isochorismate synthase (ICS) (Klessig et al., 2018). Later, IC is transported to cytoplasm by EDS5 protein, acting as transporter protein. The avr PphB Susceptible3 (PBS3) conjugates the IC and glutamate, which is further converted to SA and 3-hydroxy-acryloyl-N-glutamate (Gao et al., 2015). In PAL mediated SA biosynthesis, chorismate is converted to prephenate or phenylalanine by chorismate mutase. The PAL enzyme performs an important step to convert phenylalanine (Phe) into trans-cinnamic acid (tCA). The crucial enzyme abnormal inflorescence meristem1 (AIM1) facilitates the conversion of tCA into benzoic acid (BA), which is converted to the final product, SA by benzoic acid hydrolase (Klessig et al., 2018). In downstream signaling of SA, NPR1 activated by SA serves as coactivator of transcription of PR genes. In innate conditions, NPR1 is impounded in cytoplasm through disulfide bonds between monomers, whereas in the presence of SA, disulfide bond breaks and releases monomers that reach the nucleus to activate the transcription of PR genes (Durrant and Dong, 2004). NPR1 interacts with WRKY transcription factors, and TGA family of transcription factors facilitates their binding to the promoter region of PR genes to activate their transcription. This study reported that the genes related to SA biosynthesis, such as *PAL* & *PLD*; genes encoding for *WRKY* transcription factors (*WRKY11, WRKY31, and WRKY41*); and genes encoding PR genes *PR1* and *PR5* were highly upregulated in Cg-2 treated plants as compared to the untreated plants. It states that the SA mediated signaling pathway is completely activated from its biosynthesis to activation of defense genes. Therefore, the SAR is playing a crucial role in systemic defense induced by Cg-2 in tomato plants.

The KEGG pathway analysis stated the production of various secondary metabolites related to plant defense. Most of the secondary metabolites are synthesized through phenylpropanoid pathways. The upregulation of genes in the phenylpropanoid pathway, such as phenylalanine ammonia-lyase, p-coumaroyl CoA ligase, sinapoyl coenzyme A synthetase, feruloyl CoA ligase, caffeoyl coenzyme A synthetase, sinapoyl coenzyme A synthetase, and cinnamoyl CoA synthetase. The enhancement in the expression of genes encoding for these enzymes indicated the activation of the phenylpropanoid biosynthesis pathway that gives the most important product, lignin that has a major role in plant defense.

Earlier reports state that priming accumulates the signaling components which are highly activated on exposure to abiotic or biotic stress. The accumulation of mRNA and inactive forms of mitogen-activated protein kinases, such as *MPK3* and *MPK6* take place in primed plants (Beckers et al., 2009). When counter-inoculated by plant pathogens, MPK3 and *MPK6* are more strongly activated in primed plants as compared with non-primed plants and this is associated with increased expression of defense genes (Beckers et al., 2009). The gene modulation of MPK signaling in various phytohormone signal transduction indicated the high upregulation of *MPK3* and *MPK6* genes in tomato plants pre-inoculated with Cg-2. The increase in expression of these MPK genes mark the activation of the downstream signaling in various phytohormone signal transduction pathways.

Some of the DEGs are uncharacterized for protein function. The DEGs, such as Solyc01g005470.3, Solyc01g080870.3, and Solyc08g080650.2 showed more than three-fold upregulation in Cg-2 treated plants as compared with the control plants. These candidate genes can be characterized further for their function to decipher their role in plant defense response. The Solyc02g036370.3 gene code for protein with HTH myb-type domain is maximum upregulated up to 8.28-fold. This gene is functionally characterized as transcription factor with DNA-binding activity. This gene may act as transcription activator for different defense-related genes in plant. The Solyc03g083770.1 gene encode for protein containing PMEI domain is upregulated to 5.86s. It is characterized for pectin esterase inhibitor activity in the Uniprot database, which would inhibit the pectin esterase enzyme of pathogen. This would prevent the breakdown of plant cell wall by enzymatic attack, which is essential for direct penetration of a pathogen.

Apart from the induced resistance, tomato plant inoculation with Cg-2 enhanced the plant growth and development, which was marked by enhanced shoot and root length. This was confirmed by increase in the physiological parameters, such as photosynthesis and stomatal conductance that directly contribute to the growth and development. Further, it was supported at molecular level by an increase in the expression of genes related to cytokinin signal transduction.

*Chaetomium globosum* induces systemic resistance in the plants which is regulated by SA and JA hormone signal transduction that downstream activates the systemic defense in tomato plants. Furthermore, trisomic interaction of plant-biocontrol agent-pathogen can be visualized by transcriptome analysis of tomato plant in the presence of pathogen and biocontrol agent and the results of this study can be further validated by using mutant tomato plants with impaired hormone signaling pathways.

## Conclusion

*Chaetomium globosum* treatment induces systemic defense in tomato plants against early blight disease and enhances plant growth through augmentation in physiological processes of plant. The transcriptomic data revealed that systemic resistance involve the active participation of SA and JA hormone signaling networks, which indicate the involvement of SAR and ISR.

## Data Availability Statement

The datasets generated for this study can be found in online repositories. The names of the repository/repositories and accession number(s) can be found at: https://www.ncbi.nlm.nih.gov/, under bio-project number-PRJNA721530 with bio-sample accessions number: SAMN18719853, SAMN18719854, SAMN18719855, and SAMN18719856.

## Author Contributions

JS, RA, BB, MS, and ZH were involved in the conceptualization of the project, study design, critical inputs, and finalization of the manuscript. JS was involved in the wet lab experiments. JS, KD, PP, and AS were involved in the bio-informatics analyses and data compilation. JS, BB, and RA have drafted the manuscript. PP, BB, MS, AS, and RA edited the manuscript. All authors have read and approved the final manuscript.

## Funding

The funds for research were provided by the Director, ICAR-Indian Agricultural Research Institute, New Delhi, and the ICAR-World Bank sponsored NAHEP-CAAST project of ICAR-IARI, New Delhi.

## Conflict of Interest

The authors declare that the research was conducted in the absence of any commercial or financial relationships that could be construed as a potential conflict of interest.

## Publisher's Note

All claims expressed in this article are solely those of the authors and do not necessarily represent those of their affiliated organizations, or those of the publisher, the editors and the reviewers. Any product that may be evaluated in this article, or claim that may be made by its manufacturer, is not guaranteed or endorsed by the publisher.
