# Supplementary material for: Transcriptome Reprogramming of Tomato Orchestrate the Hormone Signaling Network of Systemic Resistance Induced by Chaetomium globosum
Source: Front Plant Sci. 2021 Sep 23;12:721193. doi: 10.3389/fpls.2021.721193 (PMC8495223; doi:10.3389/fpls.2021.721193)
Supplement: Supplementary file 1 [file Table_1.DOCX]

|  | Gene ID | Gene Name | Related pathway | Forward primer | Reverse Primer |
| --- | --- | --- | --- | --- | --- |
| 1. | K03291 | *Pi-II* | JA | GAAAATCGTTAATTTATCCCAC | ACATACAAACTTTCCATCTTTA |
| 2. | AF083253 | *MC* | JA | GAGAATTTCAAGGAAGTTCAA | GGCTTTATTTCACACAGAGATA |
| 3. | M69247 | *PR1* | SA | GTGGGATCGGATTGATATCCT | CCTAAGCCACGATACCATGAA |
| 4. | M83314 | *PAL* | SA | CGTTATGCTCTCCGAACATC | GAAGTTGCCACCATGTAAGG |
| 5. | NM001247876 | *Glu* | ET | CCATCACAGGGTTCATTTAGG | CCATCCACTCTCTGACACAACT |
| 6. | X51904 | *Le4* | ABA | ACTCAAGGCATGGGTACTGG | CCTTCTTTCTCCTCCCACCT |

**Table S1.** Primer sequences for marker genes of hormone signal transduction pathways to perform qRT-PCR
