# Supplementary material for: Transcriptome Reprogramming of Tomato Orchestrate the Hormone Signaling Network of Systemic Resistance Induced by Chaetomium globosum
Source: Front Plant Sci. 2021 Sep 23;12:721193. doi: 10.3389/fpls.2021.721193 (PMC8495223; doi:10.3389/fpls.2021.721193)
Supplement: Supplementary file 2 [file Table_2.DOCX]

**Table S2.** Primer sequences for some selected genes used for validation of results of transcriptomics data by qRT-PCR.

|  | Gene ID | Gene Name | Related pathway | Forward primer | Reverse Primer | Amplicon Size |
| --- | --- | --- | --- | --- | --- | --- |
| 1. | Solyc03g122340.2 | *LOXD* | JA | ATCCCTGACGAGAACGATCC | TCCAAGTAGACGGTTGCTGT | 178 |
| 2. | Solyc10g011660.2 | *JAR1* | JA | AATGGCACTCCAGAACTCCA | CAGCTGAGACGTTGACATGG | 172 |
| 3. | Solyc06g066370.2.1 | *WRKY31* | SA | ATGATGTTCCAGCAGCAAGG | AAGGGTGCTCCCATTTCAGA | 151 |
| 4. | Solyc08g006320.2.1 | *SlWRKY11* | SA | GTTCCGGCGATCAGTTCAAA | AGCATTCCAGGATCATCGGT | 179 |
| 5. | Solyc07g051840.2.1 | *SlWRKY41* | SA | AGGAAGGCACAGTTGCAAAG | GAATGGGAACGCCGTGAAA | 187 |
| 6. | Solyc08g080620.1.1 | *PR5* | SA | GGGTACCCGGAGGATGTAAC | CGCATTAGGGCACCTTTGTT | 122 |
| 7. | Solyc08g007820.1.1 | *ERF ½* | ET | CATCGAGCCAACCCAAGAAA | CTAGGCTCACGTAGCTCACA | 121 |
| 8. | Solyc07g009380.2.1 | *Xth2* | ET | AATGCTGAGGAATGGGCAAC | TTGGCTGTGGGATTCTTGGA | 116 |
| 9. | Solyc10g085310.1.1 | *PYL4* | ABA | ACTTTACGGGAAGTCCGTGT | GTTCCGTGTGAAGCGTAGTC | 160 |
| 10. | Solyc02g085020.2.1 | *DFR* | Phenylpropanoid | CCATTGAGACTTGCCGACAG | ACAATTGGCAACTGGTGCAT | 140 |
| 11. | Solyc02g092580.2.1 | *Peroxidase* | Defense related | AGGGAACACAGCTGAGAAGG | TCTGGTGGCTAAAGCGAGAA | 151 |
| 12. | Solyc10g055800.1.1 | *Chitinase* | Defense related | ATGTGGTAGACAGGCAGGAG | AGGTTTCGGAGAAGGACCAG | 139 |
