# Supplementary material for: Transcriptome Reprogramming of Tomato Orchestrate the Hormone Signaling Network of Systemic Resistance Induced by Chaetomium globosum
Source: Front Plant Sci. 2021 Sep 23;12:721193. doi: 10.3389/fpls.2021.721193 (PMC8495223; doi:10.3389/fpls.2021.721193)
Supplement: Supplementary file 3 [file Table_3.DOCX]

**Table S3.** Statistics of sequencing data for untreated plant and Cg-2 treated plant

|  |  | Raw sequencing data | | | Sequencing data after trimming | | |
| --- | --- | --- | --- | --- | --- | --- | --- |
| **Sr. No.** | **Sample** | **No. of**  **Reads** | **Read length** | **GC%** | **No. of**  **Reads** | **Read length** | **GC%** |
| 1. | Untreated_R1 | 20003620 | 150 | 44 | 19489448 | 150 | 42 |
| 2. | Untreated_R2 | 20546374 | 150 | 45 | 19865441 | 150 | 43 |
| 3. | Treated_R1 | 20009074 | 150 | 51 | 18638910 | 150 | 49 |
| 4. | Treated_R2 | 20985360 | 150 | 44 | 20199066 | 150 | 42 |
