# Supplementary material for: Transcriptome Reprogramming of Tomato Orchestrate the Hormone Signaling Network of Systemic Resistance Induced by Chaetomium globosum
Source: Front Plant Sci. 2021 Sep 23;12:721193. doi: 10.3389/fpls.2021.721193 (PMC8495223; doi:10.3389/fpls.2021.721193)
Supplement: Supplementary file 4 [file Table_4.DOCX]

**Table S4**. The top 50 DEGs upregulated in tomato plant in response to *Chaetomium globosum* Cg-2 treatment

| **Sr. No** | **Gene id** | **Gene** | **Fold change** | | **Protein name** | **Gene Ontology** |
| --- | --- | --- | --- | --- | --- | --- |
| 1. | XLOC_031651 | Solyc09g010980.1 | inf | Uncharacterized protein | | nucleus [GO:0005634]; negative regulation of cell cycle [GO:0045786]; negative regulation of mitotic nuclear division [GO:0045839]; regulation of DNA endoreduplication [GO:0032875] |
| 2. | XLOC_000424 | Solyc01g017140.1 | inf | ATPase_AAA_core domain-containing protein | | chloroplast [GO:0009507]; ATP binding [GO:0005524] |
| 3. | XLOC_018747 | Solyc04g039840.1 | inf | Ribulose bisphosphate carboxylase large chain (EC 4.1.1.39) | | plastid [GO:0009536]; monooxygenase activity [GO:0004497]; ribulose-bisphosphate carboxylase activity [GO:0016984]; photorespiration [GO:0009853]; reductive pentose-phosphate cycle [GO:0019253] |
| 4. | XLOC_030332 | Solyc08g007830.1 | inf | AP2/ERF domain-containing protein | | nucleus [GO:0005634]; DNA binding [GO:0003677]; DNA-binding transcription factor activity [GO:0003700]; defense response [GO:0006952] |
| 5. | XLOC_013553 | Solyc02g036370.3 | 8.28775 | HTH myb-type domain-containing protein | | nucleus [GO:0005634] |
| 6. | XLOC_018512 | Solyc04g010250.3 | 7.57587 | Hydrolase_4 domain-containing protein | | membrane [GO:0016020]; lipase activity [GO:0016298] |
| 7. | XLOC_002084 | Solyc01g005470.3 | 6.31669 | Uncharacterized protein | | integral component of membrane [GO:0016021] |
| 8. | XLOC_015760 | Solyc03g083770.1 | 5.86016 | PMEI domain-containing protein | | extracellular region [GO:0005576]; enzyme inhibitor activity [GO:0004857]; hydrolase activity [GO:0016787]; negative regulation of catalytic activity [GO:0043086] |
| 9. | XLOC_003133 | Solyc01g080870.3 | 5.70676 | Uncharacterized protein | | integral component of membrane [GO:0016021]; transmembrane transporter activity [GO:0022857]; phosphate ion transport [GO:0006817] |
| 10. | XLOC_005703 | Solyc10g009240.3 | 5.4506 | 3-ketoacyl-CoA synthase (EC 2.3.1.-) | | integral component of membrane [GO:0016021]; 3-oxo-arachidoyl-CoA synthase activity [GO:0102336]; 3-oxo-cerotoyl-CoA synthase activity [GO:0102337]; 3-oxo-lignoceronyl-CoA synthase activity [GO:0102338]; very-long-chain 3-ketoacyl-CoA synthase activity [GO:0102756]; fatty acid biosynthetic process [GO:0006633] |
| 11. | XLOC_026380 | Solyc06g083650.3 | 5.38058 | Lipase_GDSL domain-containing protein | | hydrolase activity, acting on ester bonds [GO:0016788] |
| 12. | XLOC_000015 | Solyc01g005300.3 | 5.37737 | PAS domain-containing protein | | cytosol [GO:0005829]; nucleus [GO:0005634]; SCF ubiquitin ligase complex [GO:0019005]; photoreceptor activity [GO:0009881]; circadian rhythm [GO:0007623]; protein ubiquitination [GO:0016567]; protein-chromophore linkage [GO:0018298]; response to blue light [GO:0009637] |
| 13. | XLOC_029071 | Solyc08g007130.3 | 5.11916 | Beta-amylase (EC 3.2.1.2) | | amylopectin maltohydrolase activity [GO:0102229]; beta-amylase activity [GO:0016161]; polysaccharide catabolic process [GO:0000272] |
| 14. | XLOC_030371 | Solyc08g008485.1 | 5.09155 | Uncharacterized protein | | nucleus [GO:0005634]; DNA binding [GO:0003677] |
| 15. | XLOC_030941 | Solyc08g068770.2 | 5.03385 | N-acetyltransferase domain-containing protein | | N-acetyltransferase activity [GO:0008080] |
| 16. | XLOC_010359 | Solyc12g099940.2 | 5.03128 | N-acetyltransferase domain-containing protein | | N-acetyltransferase activity [GO:0008080] |
| 17. | XLOC_000705 | Solyc01g059965.1 | 5.01031 | Glucan endo-1,3-beta-glucosidase B (EC 3.2.1.39) ((1->3)-beta-glucan endohydrolase B) ((1->3)-beta-glucanase B) (Basic beta-1,3-glucanase) (Beta-1,3-endoglucanase B) | | anchored component of plasma membrane [GO:0046658]; vacuole [GO:0005773]; glucan endo-1,3-beta-D-glucosidase activity [GO:0042973]; carbohydrate metabolic process [GO:0005975]; defense response [GO:0006952] |
| 18. | XLOC_016055 | Solyc03g111710.3 | 4.99622 | BTB domain-containing protein | | nucleus [GO:0005634]; metal ion binding [GO:0046872] |
| 19. | XLOC_009994 | Solyc12g045030.2 | 4.94278 | Uncharacterized protein | | oxidoreductase activity [GO:0016491] |
| 20. | XLOC_008622 | Solyc11g044910.2 | 4.93409 | Fn3_like domain-containing protein | | extracellular region [GO:0005576]; plant-type cell wall [GO:0009505]; alpha-L-arabinofuranosidase activity [GO:0046556]; hydrolase activity, hydrolyzing O-glycosyl compounds [GO:0004553]; xylan 1,4-beta-xylosidase activity [GO:0009044]; arabinan catabolic process [GO:0031222]; xylan catabolic process [GO:0045493] |
| 21. | XLOC_000913 | Solyc01g079740.3 | 4.861 | Uncharacterized protein | | - |
| 22. | XLOC_023304 | Solyc05g052040.1 | 4.7916 | AP2/ERF domain-containing protein | | nucleus [GO:0005634]; DNA binding [GO:0003677]; DNA-binding transcription factor activity [GO:0003700]; defense response [GO:0006952] |
| 23. | XLOC_002127 | Solyc01g006300.3 | 4.6397 | Peroxidase (EC 1.11.1.7) | | extracellular region [GO:0005576]; heme binding [GO:0020037]; metal ion binding [GO:0046872]; peroxidase activity [GO:0004601]; hydrogen peroxide catabolic process [GO:0042744]; response to oxidative stress [GO:0006979] |
| 24. | XLOC_005424 | Solyc10g085310.1 | 4.4939 | Uncharacterized protein | | cytoplasm [GO:0005737]; nucleus [GO:0005634]; plasma membrane [GO:0005886]; abscisic acid binding [GO:0010427]; protein phosphatase inhibitor activity [GO:0004864]; signaling receptor activity [GO:0038023]; abscisic acid-activated signaling pathway [GO:0009738]; regulation of protein serine/threonine phosphatase activity [GO:0080163] |
| 25. | XLOC_023692 | Solyc06g009140.3 | 4.4894 | Uncharacterized protein | | mitochondrion [GO:0005739] |
| 26. | XLOC_026195 | Solyc06g074800.1 | 4.38922 | Uncharacterized protein | | - |
| 27. | XLOC_030331 | Solyc08g007820.1 | 4.38021 | AP2/ERF domain-containing protein | | nucleus [GO:0005634]; DNA binding [GO:0003677]; DNA-binding transcription factor activity [GO:0003700]; defense response [GO:0006952] |
| 28. | XLOC_019114 | Solyc04g056340.3 | 4.37158 | Uncharacterized protein | | cytoplasm [GO:0005737]; integral component of membrane [GO:0016021]; protein tyrosine phosphatase activity [GO:0004725] |
| 29. | XLOC_028500 | Solyc07g052250.3 | 4.34945 | Uncharacterized protein | | - |
| 30. | XLOC_015912 | Solyc03g097170.3 | 4.29897 | 3Beta_HSD domain-containing protein | | 3-beta-hydroxy-delta5-steroid dehydrogenase activity [GO:0003854]; oxidoreductase activity [GO:0016491]; oxidoreductase activity, acting on the CH-OH group of donors, NAD or NADP as acceptor [GO:0016616]; steroid biosynthetic process [GO:0006694] |
| 31. | XLOC_027639 | Solyc07g065600.3 | 4.27682 | INCENP_ARK-bind domain-containing protein | | cytoplasm [GO:0005737]; nucleus [GO:0005634]; spindle [GO:0005819] |
| 32. | XLOC_013495 | Solyc02g032360.3 | 4.2435 | - | | - |
| 33. | XLOC_009661 | Solyc12g070220.2 | 4.21471 | Uncharacterized protein | | chloroplast [GO:0009507]; ATP binding [GO:0005524] |
| 34. | XLOC_022028 | Solyc05g052050.1 | 4.1744 | AP2/ERF domain-containing protein | | nucleus [GO:0005634]; DNA binding [GO:0003677]; DNA-binding transcription factor activity [GO:0003700]; defense response [GO:0006952] |
| 35. | XLOC_008179 | Solyc11g010730.2 | 4.11249 | Protein kinase domain-containing protein | | ATP binding [GO:0005524]; protein kinase activity [GO:0004672] |
| 36. | XLOC_007425 | Solyc11g045440.1 | 4.10843 | Uncharacterized protein | | - |
| 37. | XLOC_026665 | Solyc07g009380.3 | 4.06793 | Xyloglucan endotransglucosylase/hydrolase (EC 2.4.1.207) | | apoplast [GO:0048046]; cell wall [GO:0005618]; hydrolase activity, hydrolyzing O-glycosyl compounds [GO:0004553]; xyloglucan:xyloglucosyl transferase activity [GO:0016762]; cell wall biogenesis [GO:0042546]; cell wall organization [GO:0071555]; xyloglucan metabolic process [GO:0010411] |
| 38. | XLOC_018200 | Solyc03g121090.3 | 4.06484 | Uncharacterized protein | | - |
| 39. | XLOC_016045 | Solyc03g111385.1 | 4.06207 | Uncharacterized protein | | - |
| 40. | XLOC_021911 | Solyc05g049990.3 | 4.05191 | Uncharacterized protein | | - |
| 41. | XLOC_002888 | Solyc01g065700.3 | 4.00401 | PPM-type phosphatase domain-containing protein | | cytosol [GO:0005829]; nucleus [GO:0005634]; protein serine/threonine phosphatase activity [GO:0004722] |
| 42. | XLOC_007115 | Solyc11g012690.2 | 3.99344 | Uncharacterized protein | | metal ion binding [GO:0046872]; metal ion transport [GO:0030001] |
| 43. | XLOC_009182 | Solyc12g006050.2 | 3.97528 | Uncharacterized protein | | integral component of membrane [GO:0016021]; nitrate transmembrane transporter activity [GO:0015112]; phosphate ion transport [GO:0006817]; response to nitrate [GO:0010167] |
| 44. | XLOC_024417 | Solyc06g066370.3 | 3.9441 | Uncharacterized protein | | nucleus [GO:0005634]; DNA-binding transcription factor activity [GO:0003700]; sequence-specific DNA binding [GO:0043565] |
| 45. | XLOC_012913 | Solyc02g088070.3 | 3.91203 | Dof-type domain-containing protein | | DNA binding [GO:0003677]; DNA-binding transcription factor activity [GO:0003700] |
| 46. | XLOC_012913 | Solyc02g088075.1 | 3.91203 | Uncharacterized protein | | - |
| 47. | XLOC_003565 | Solyc01g097270.3 | 3.87672 | Uncharacterized protein | | chitin binding [GO:0008061]; defense response to bacterium [GO:0042742]; defense response to fungus [GO:0050832] |
| 48. | XLOC_008409 | Solyc11g020670.1 | 3.87207 | TCP domain-containing protein | | nucleus [GO:0005634]; DNA-binding transcription factor activity [GO:0003700]; sequence-specific DNA binding [GO:0043565] |
| 49. | XLOC_023682 | Solyc06g008890.3 | 3.86682 | Protein kinase domain-containing protein | | integral component of membrane [GO:0016021]; ATP binding [GO:0005524]; protein serine/threonine kinase activity [GO:0004674] |
| 50. | XLOC_014921 | Solyc02g094190.3 | 3.85591 | Nodulin-like domain-containing protein | | integral component of membrane [GO:0016021]; membrane [GO:0016020]; phosphate ion transport [GO:0006817] |
