# Supplementary figures and images for: Transcriptome Reprogramming of Tomato Orchestrate the Hormone Signaling Network of Systemic Resistance Induced by Chaetomium globosum
Source: Front Plant Sci. 2021 Sep 23;12:721193. doi: 10.3389/fpls.2021.721193 (PMC8495223; doi:10.3389/fpls.2021.721193)

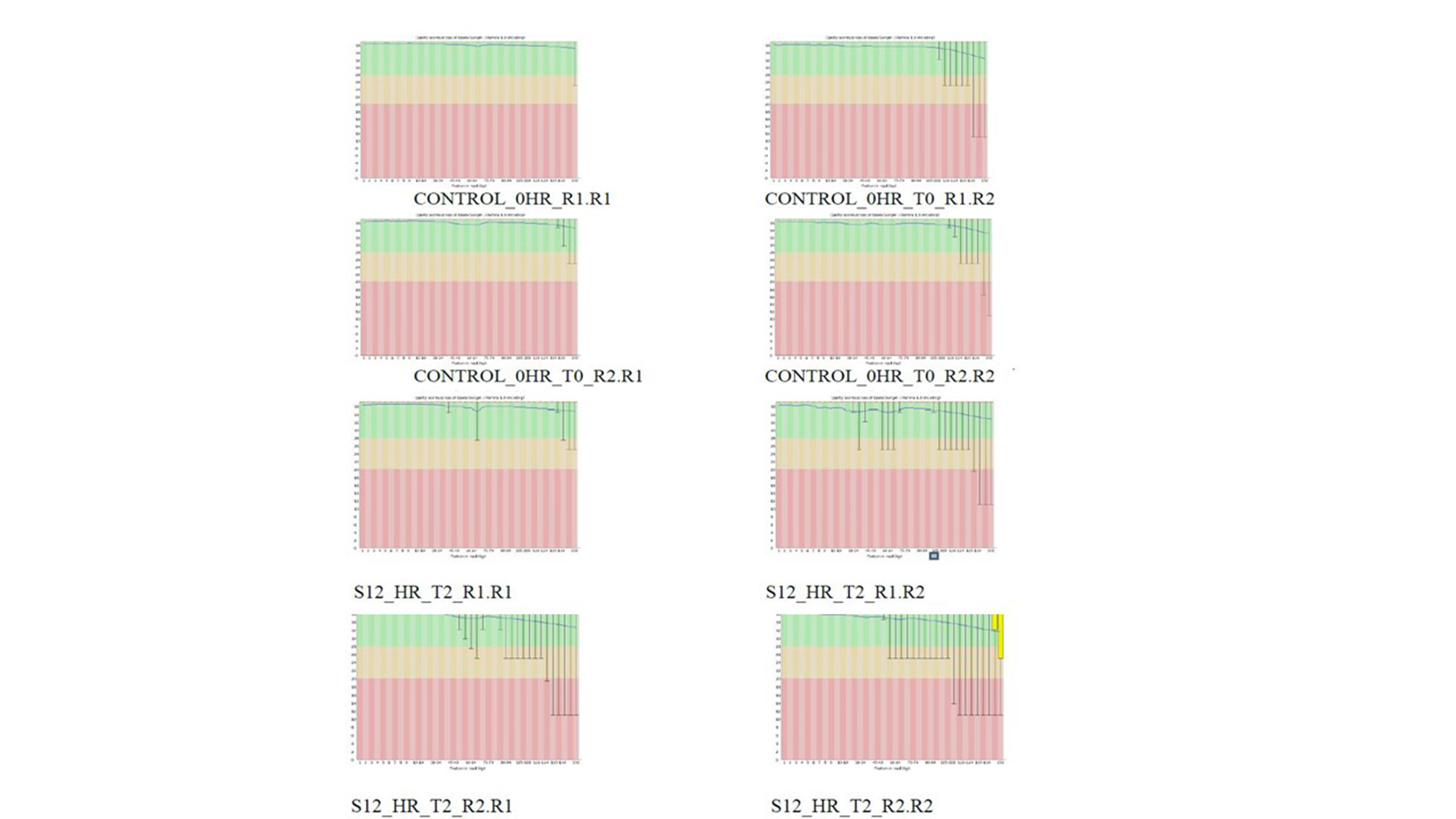

Supplement: Supplementary file 8 [file Image_1.jpg]

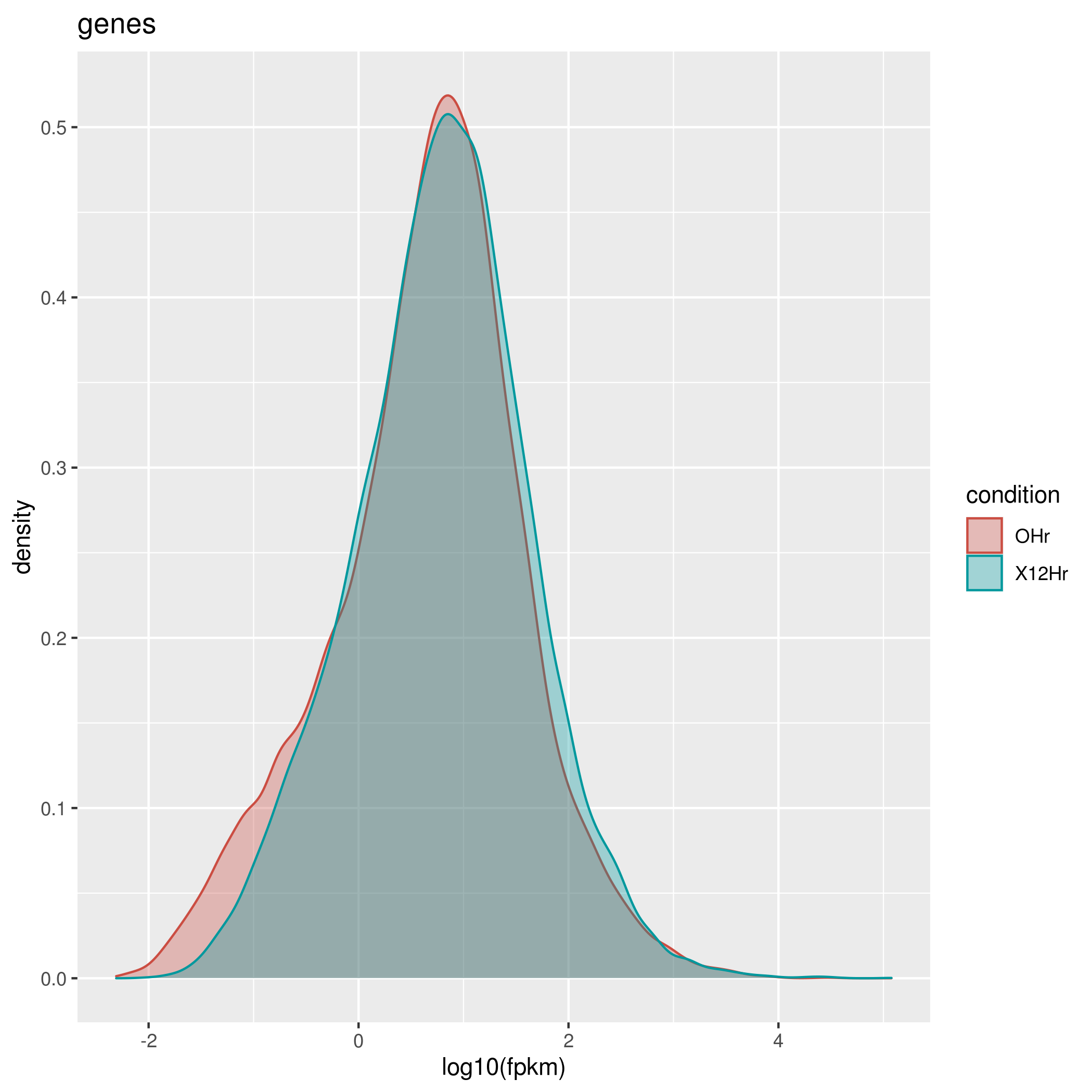

Supplement: Supplementary file 9 [file Image_2.PNG]

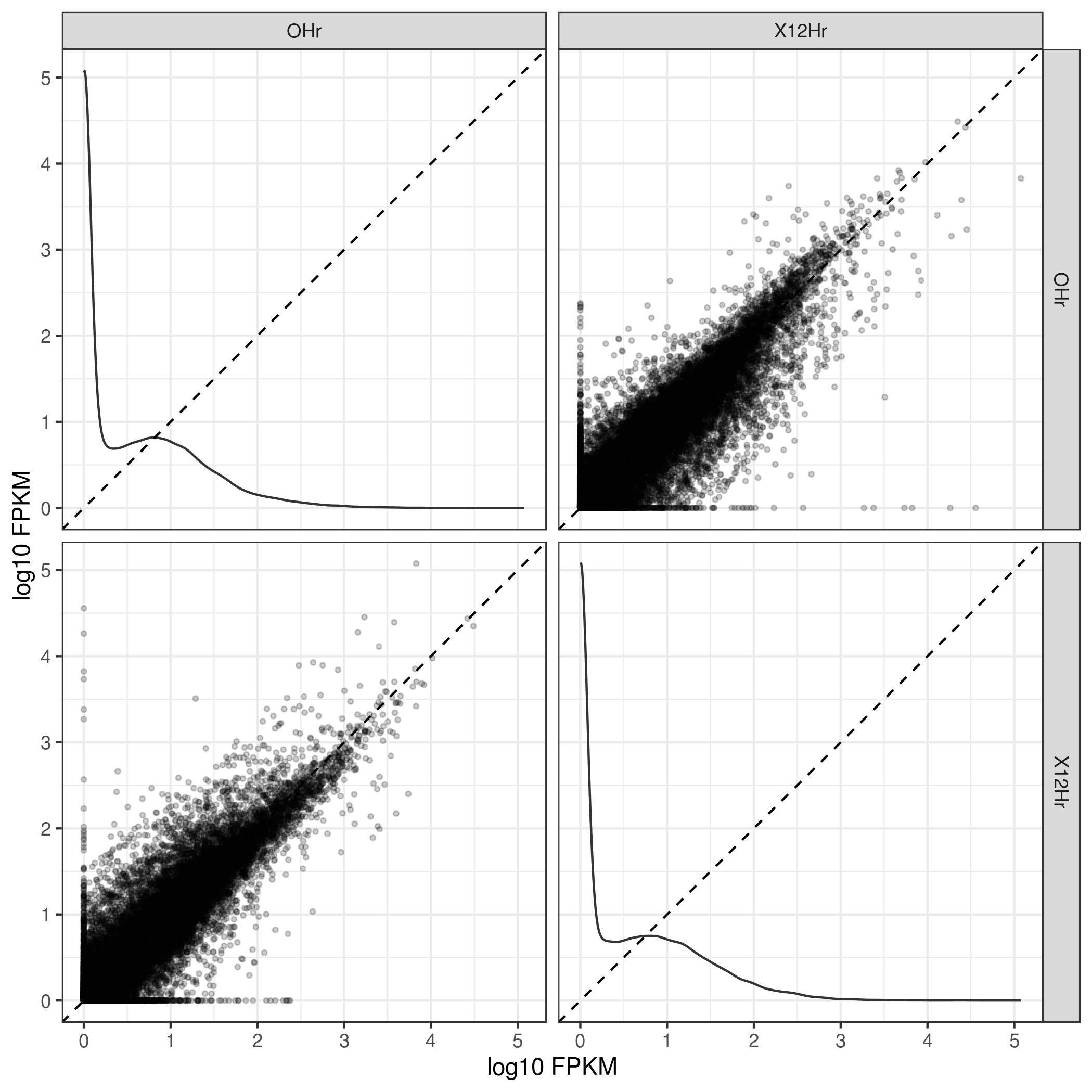

Supplement: Supplementary file 10 [file Image_3.PNG]

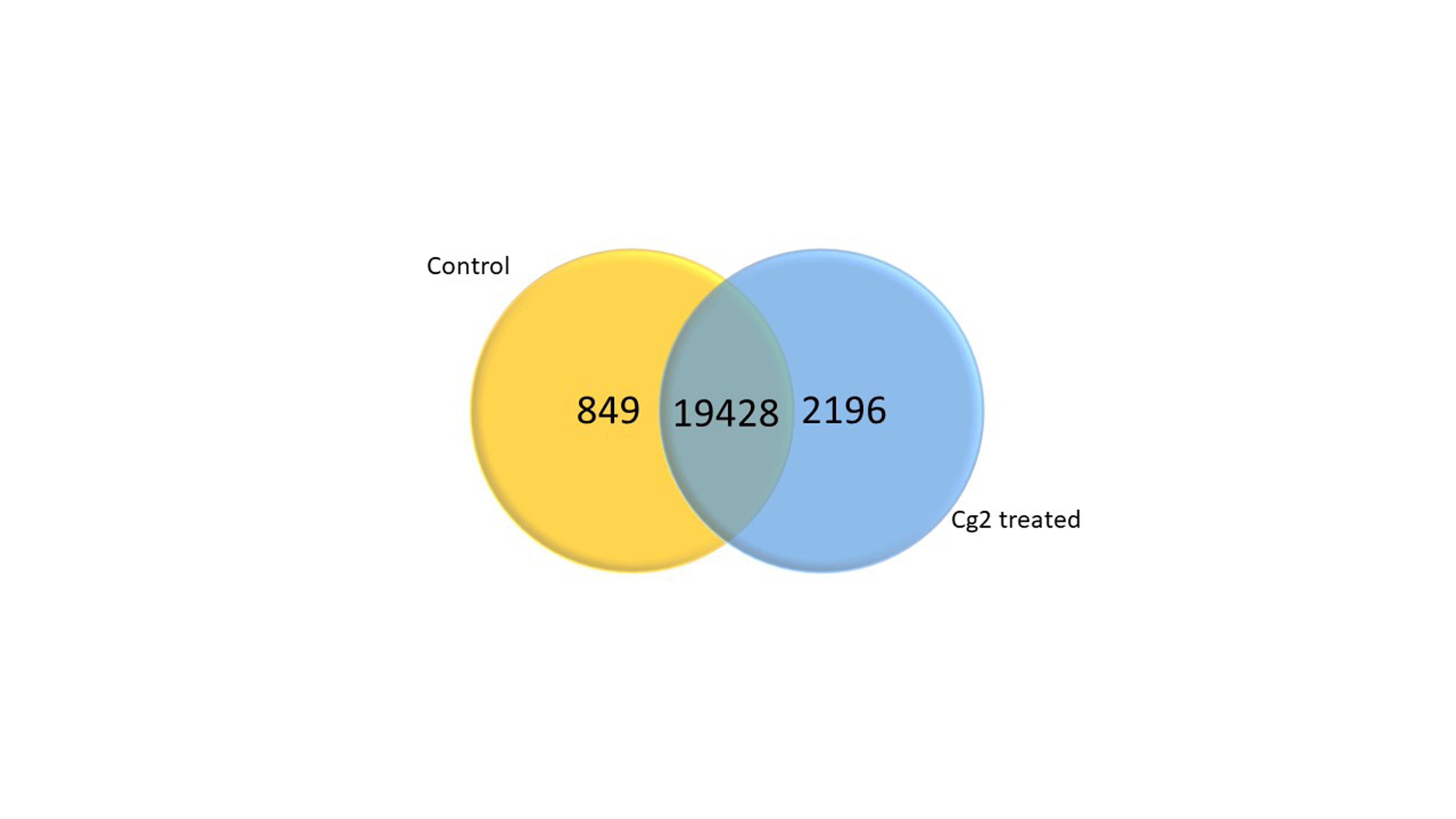

Supplement: Supplementary file 11 [file Image_4.jpg]

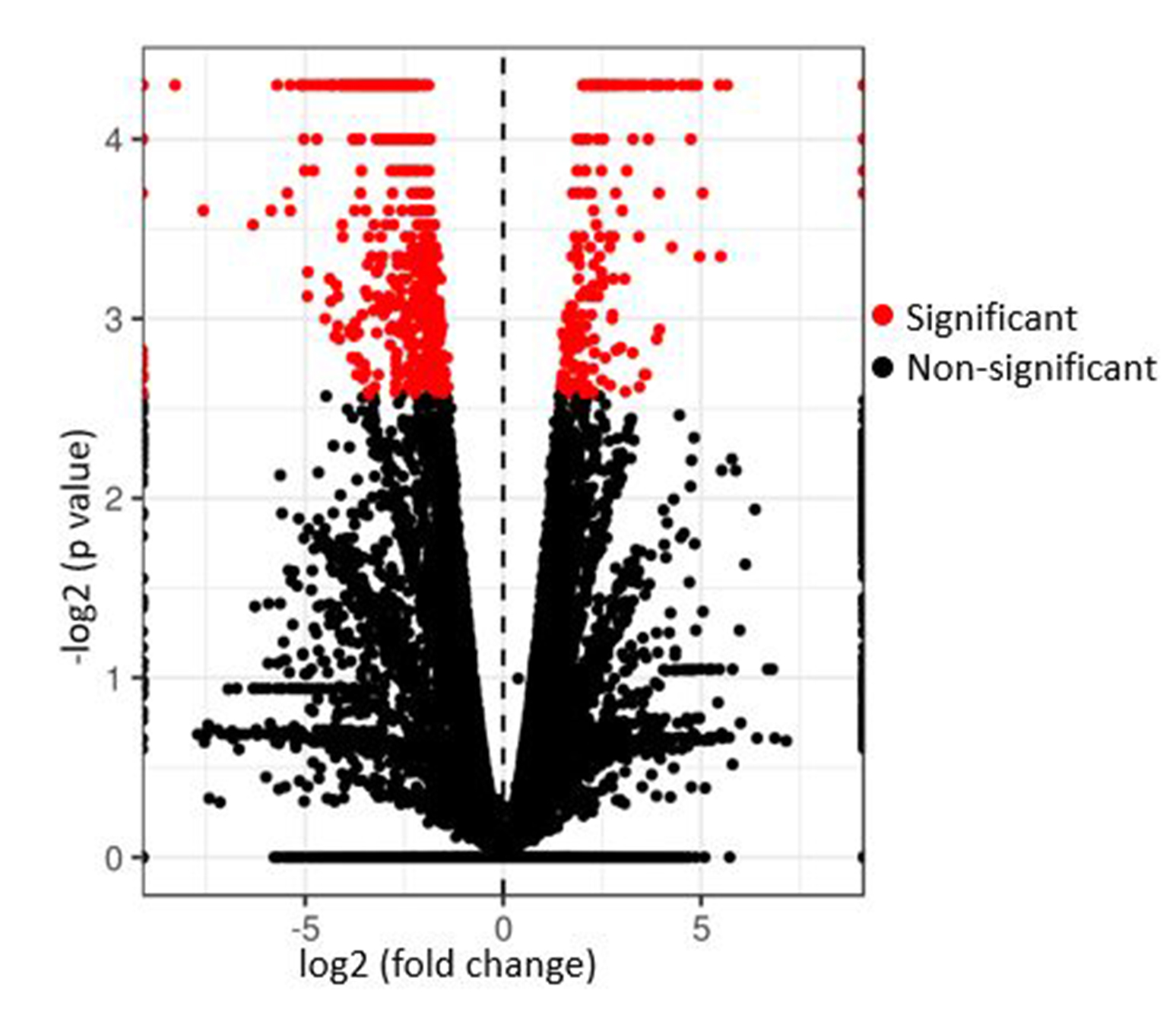

Supplement: Supplementary file 12 [file Image_5.JPEG]
